# Supplementary material for: Selective targeting of IRAK1 attenuates low molecular weight hyaluronic acid-induced stemness and non-canonical STAT3 activation in epithelial ovarian cancer
Source: Cell Death Dis. 2024 May 25;15(5):362. doi: 10.1038/s41419-024-06717-3 (PMC11127949; doi:10.1038/s41419-024-06717-3)
Supplement: Supplementary file 1 — Uncropped Western blots [file 41419_2024_6717_MOESM1_ESM.pdf]

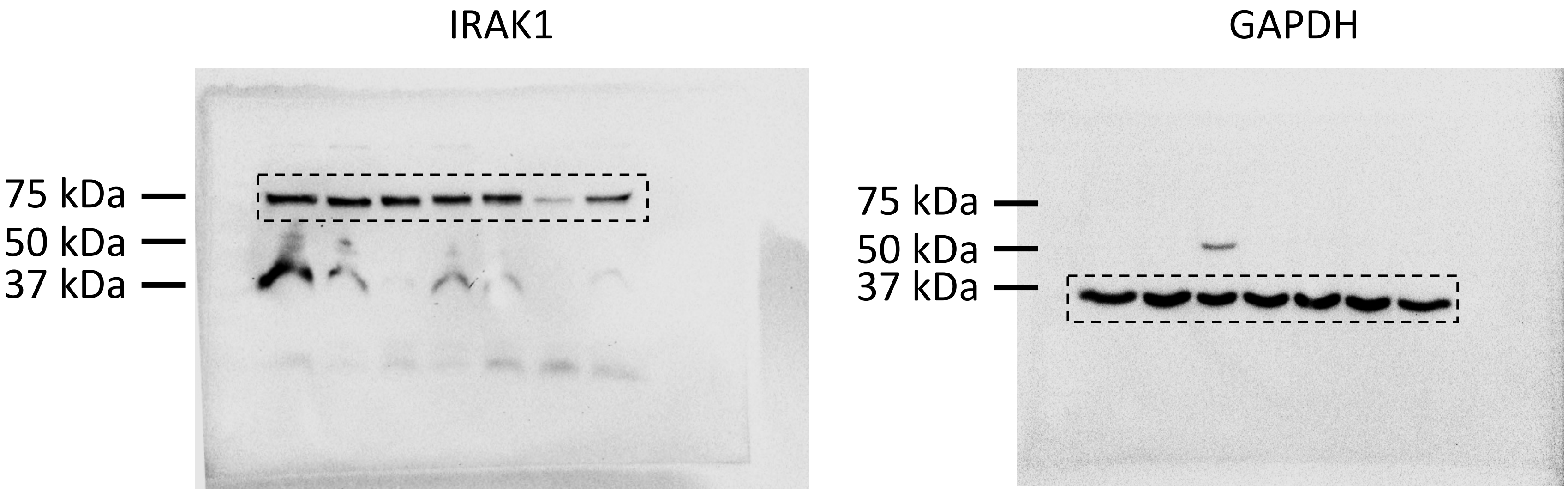


Figure 3A

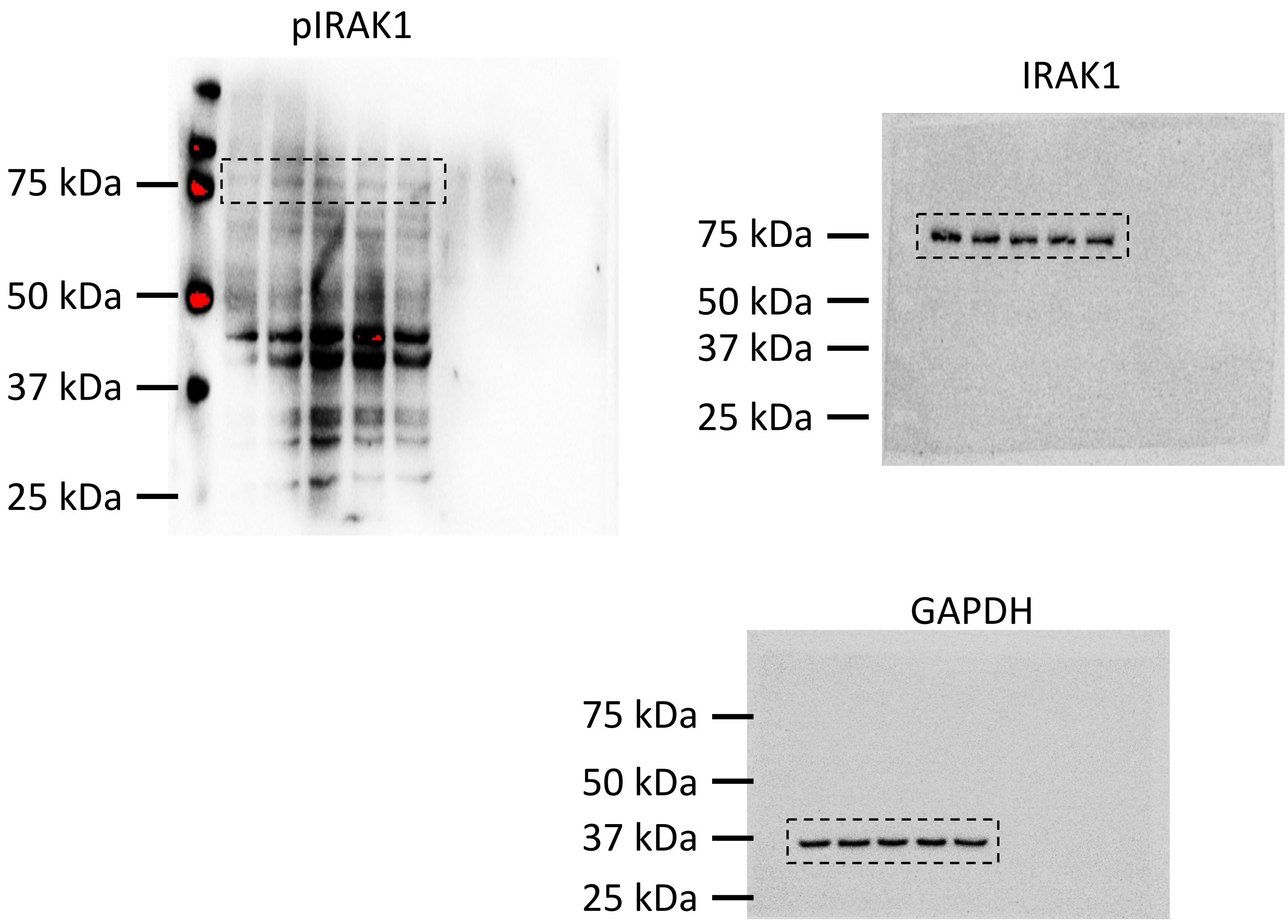


Figure 3C-A1847

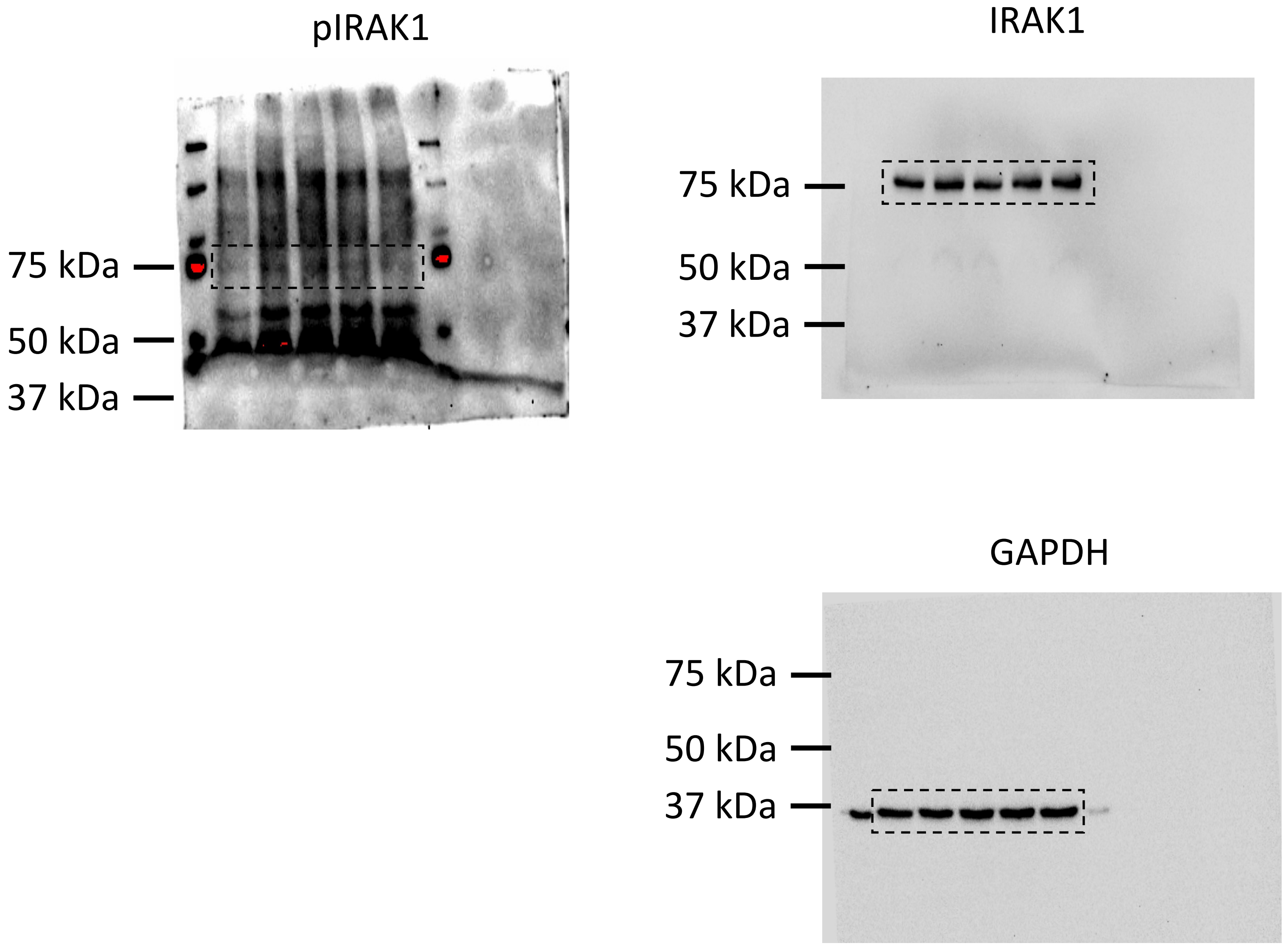


Figure 3C-OVCAR8

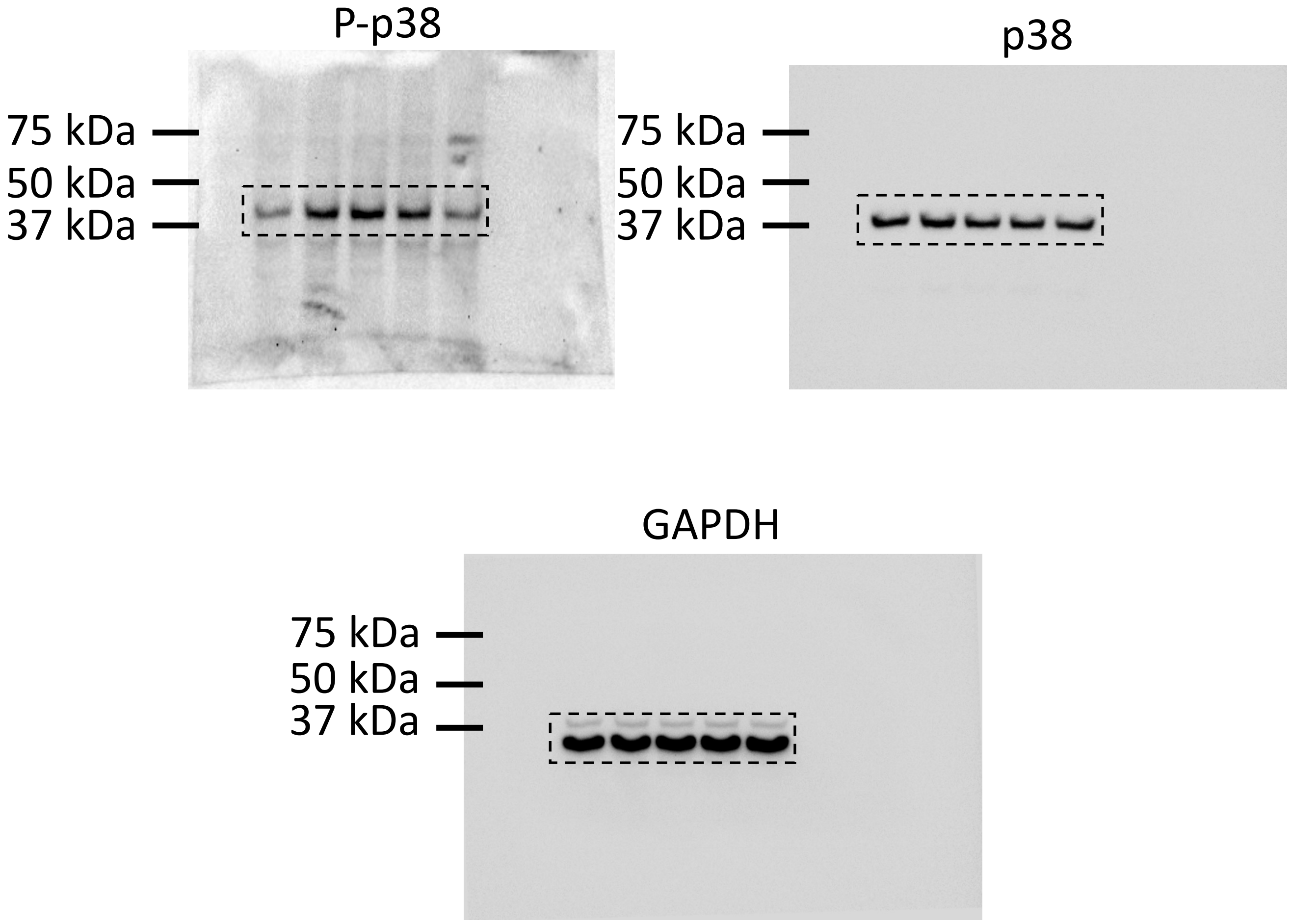


Figure 3G-A1847

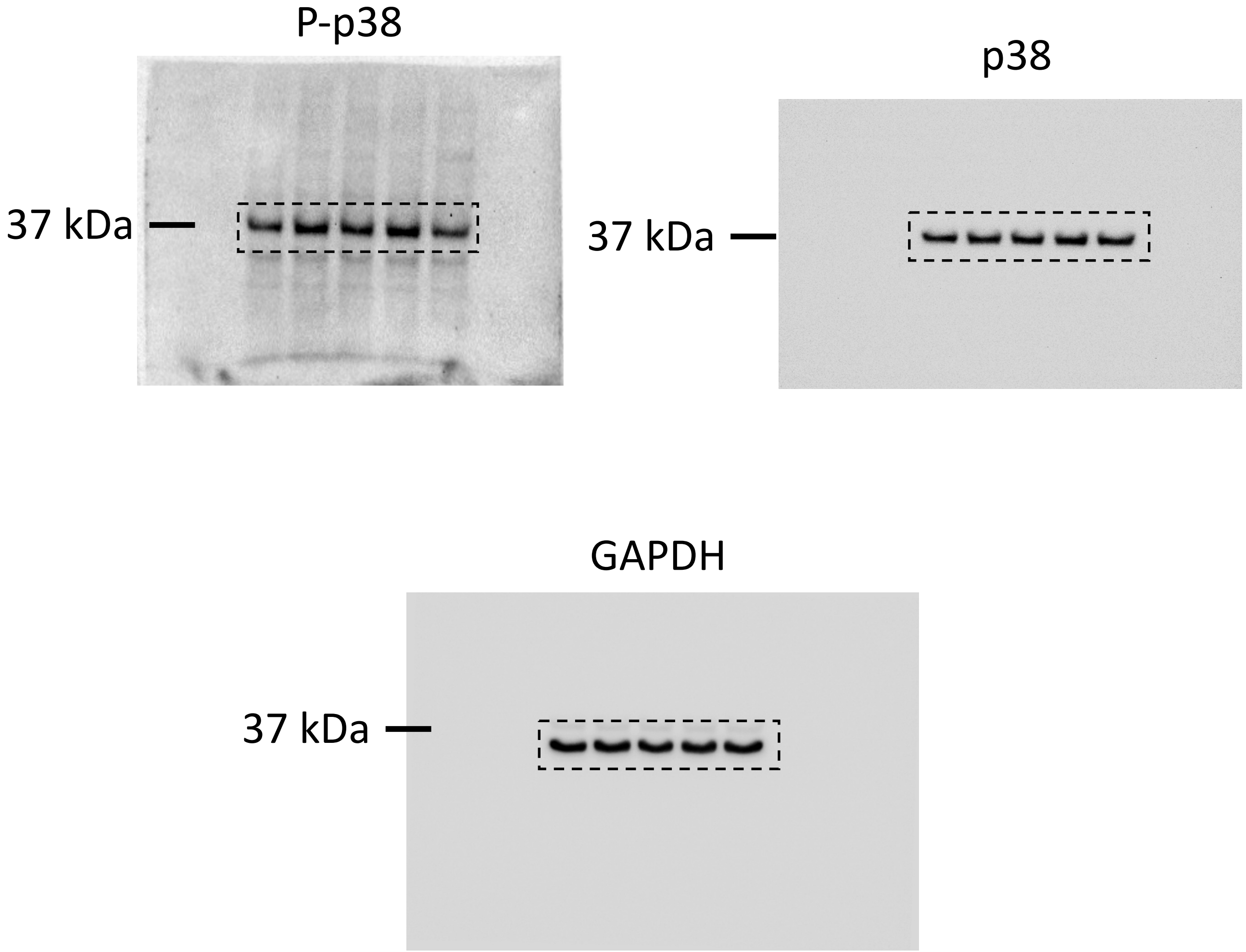


Figure 3G-OVCAR8

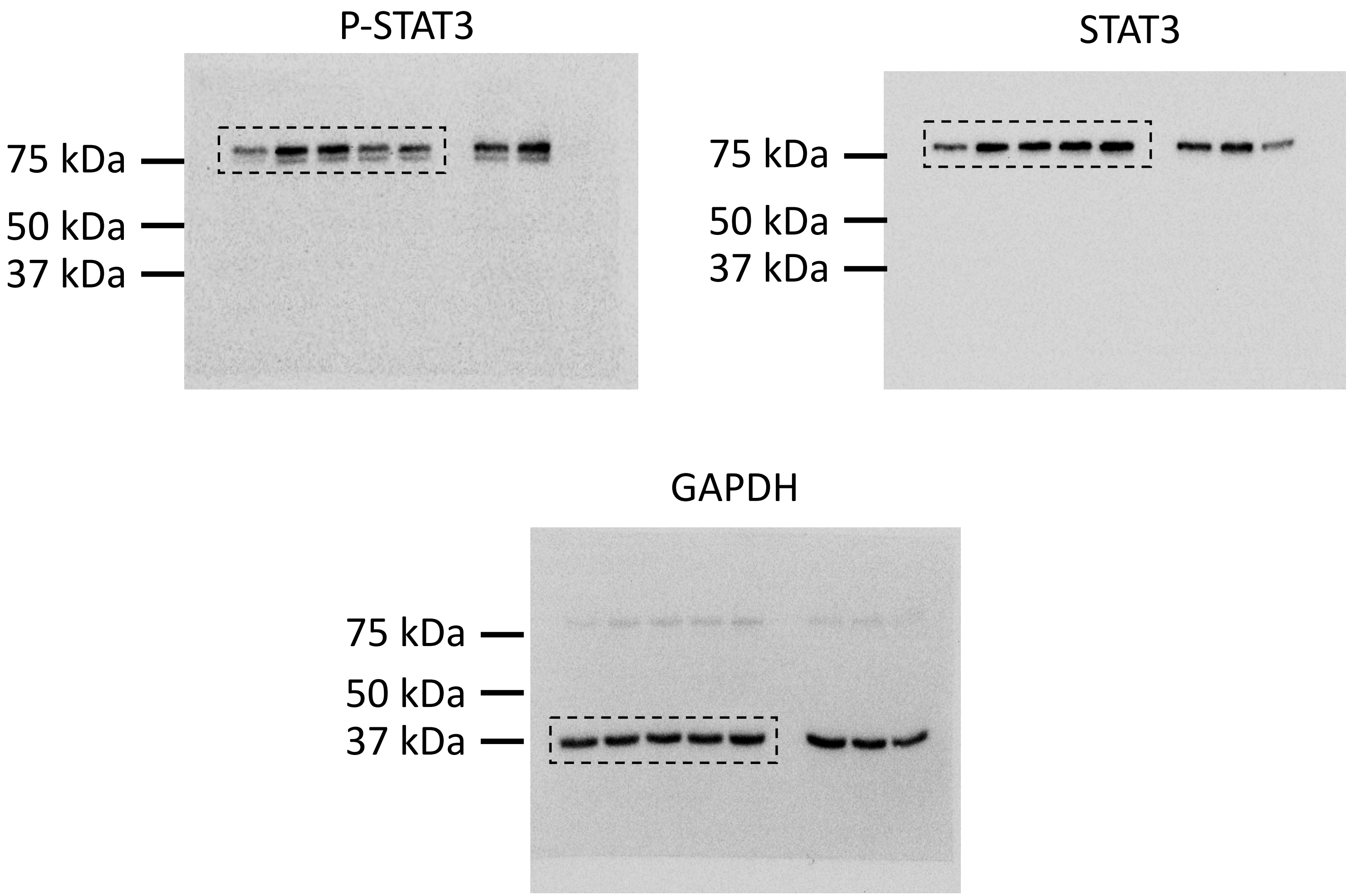


Figure 3G-A1847

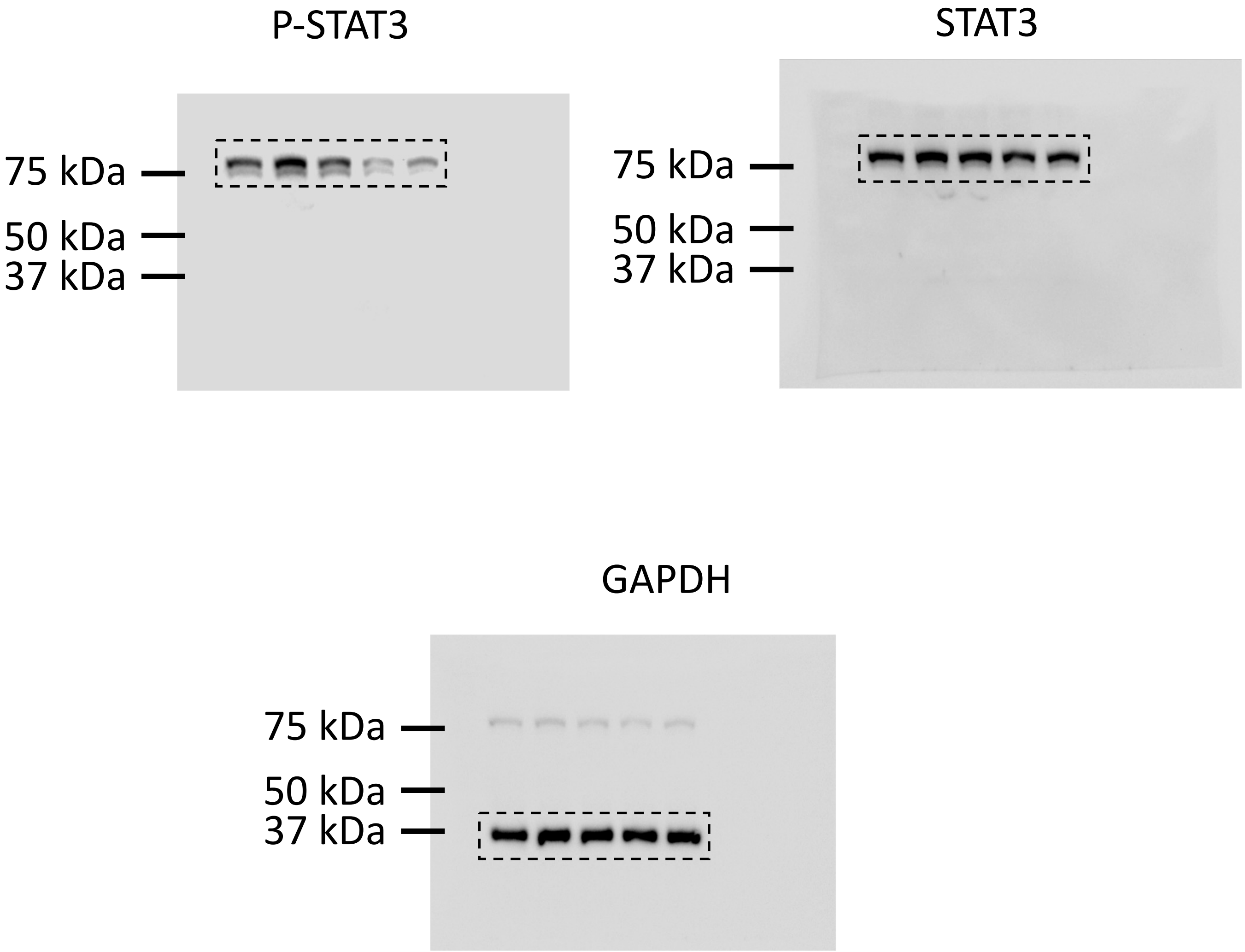


Figure 3G-OVCAR8

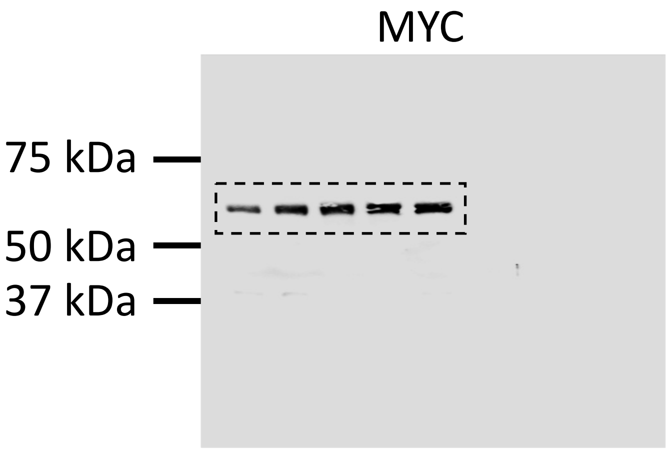


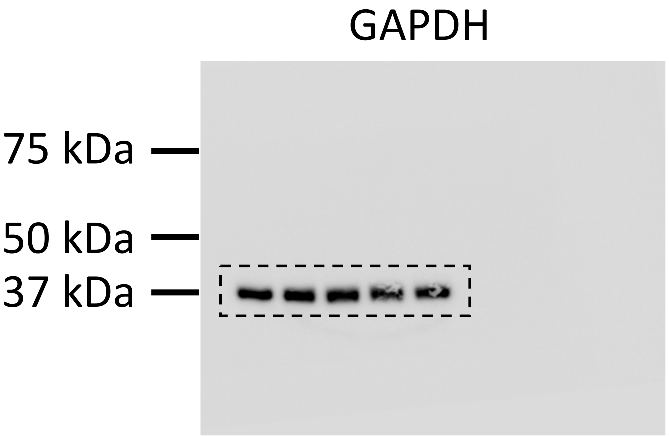


Figure 3G-OVCAR8

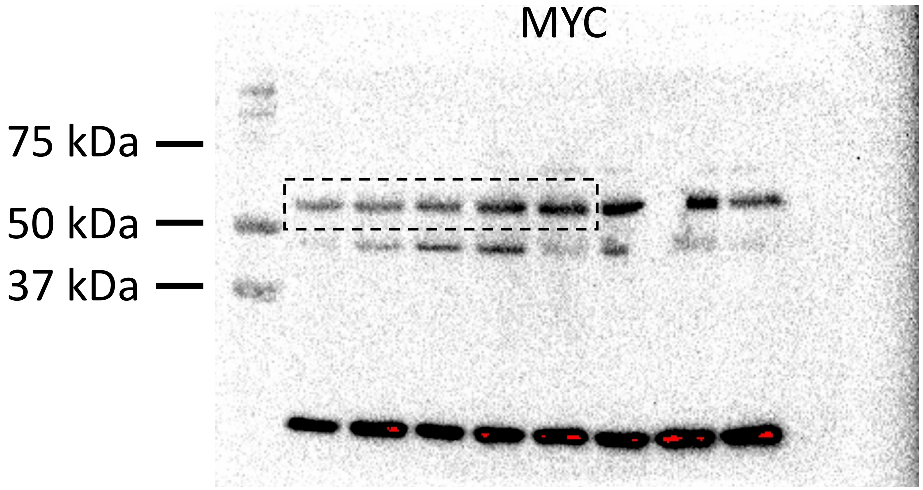


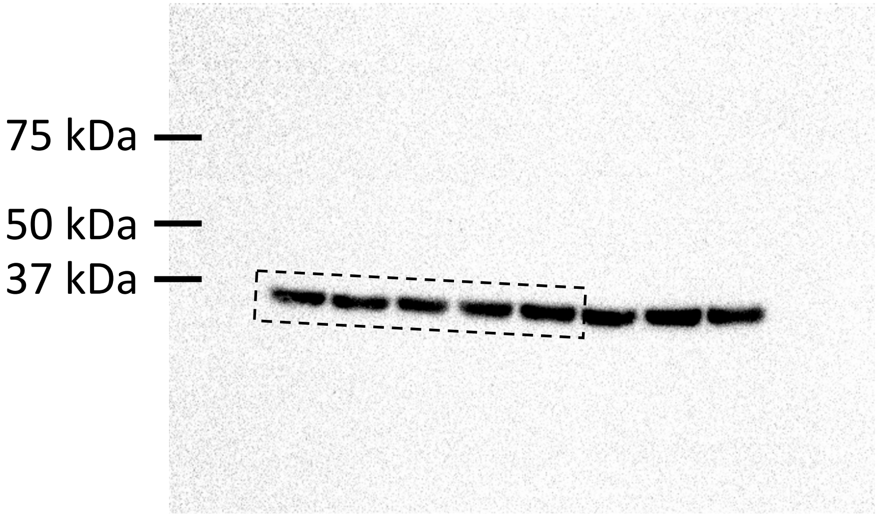


Figure 3G-A1847

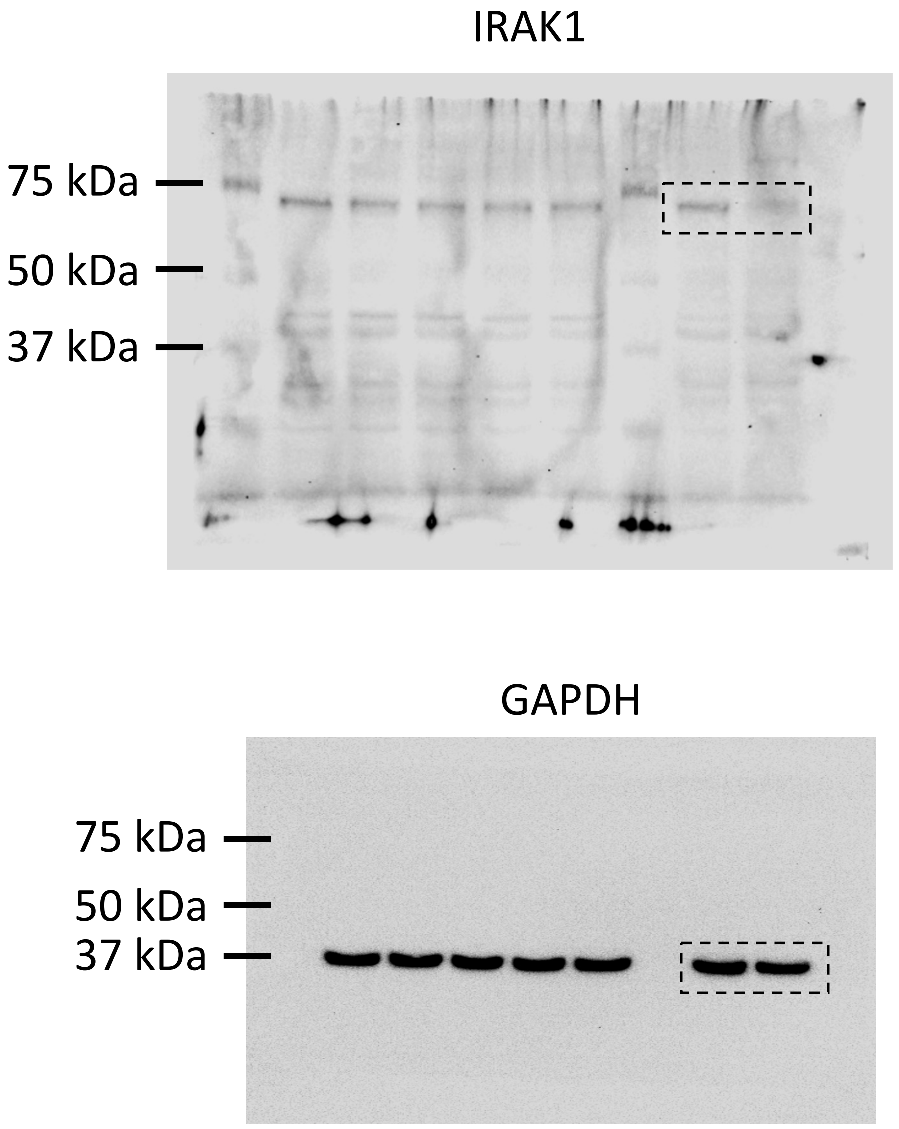


Figure 4A

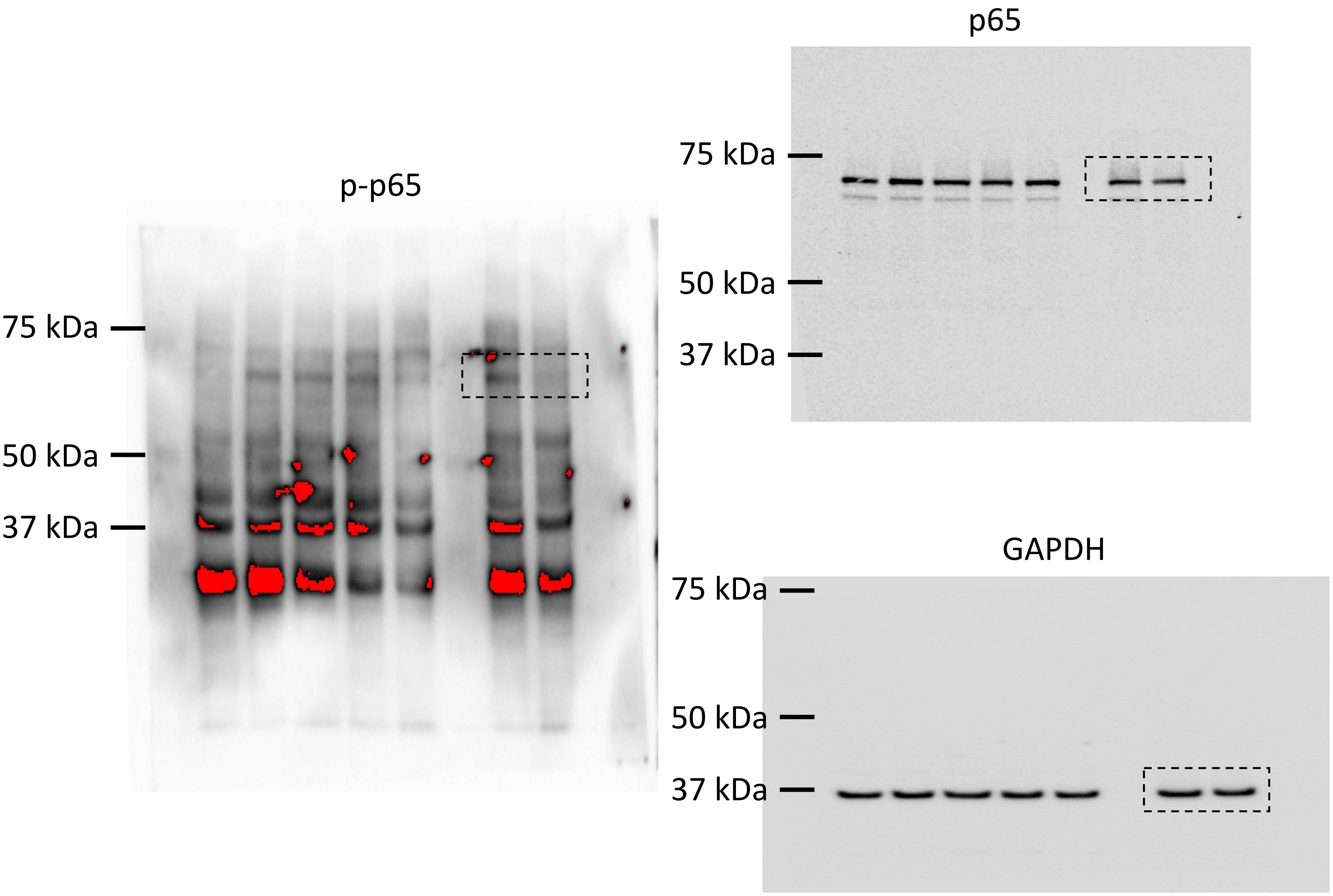
p-p65
75 kDa
50 kDa
37 kDa
p65
75 kDa
50 kDa
37 kDa
GAPDH
75 kDa
50 kDa
37 kDa

Figure 4A

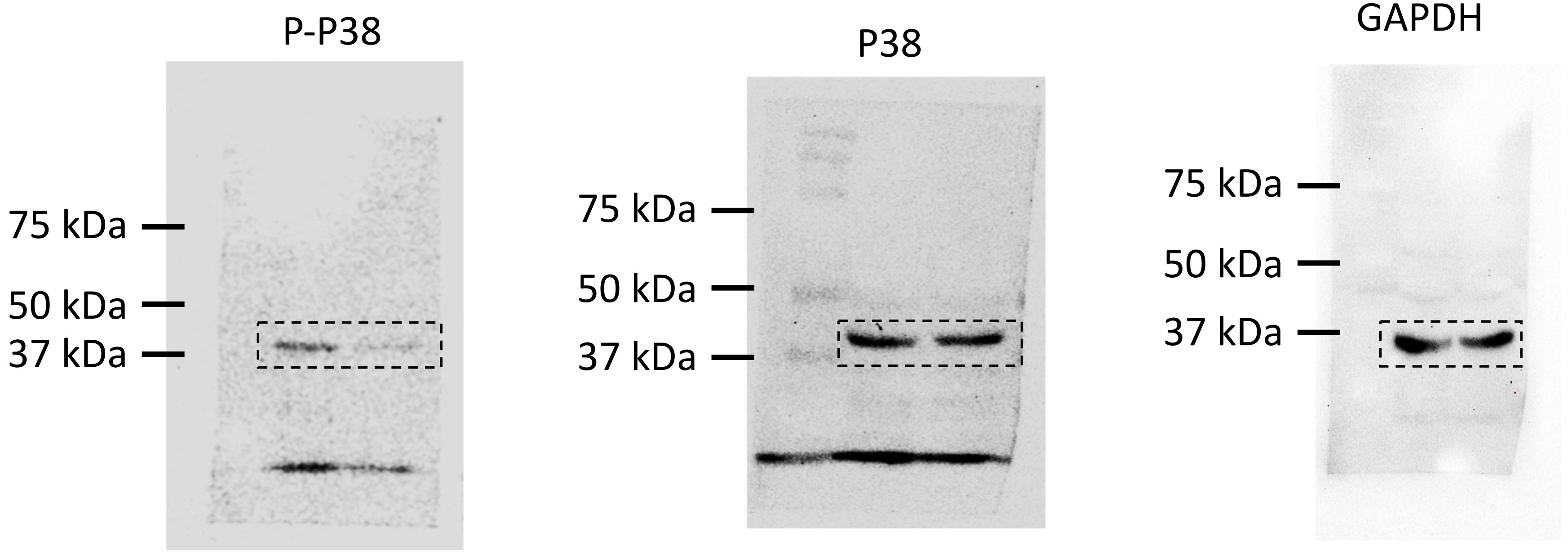
P-P38
75 kDa
50 kDa
37 kDa
P38
75 kDa
50 kDa
37 kDa
GAPDH
75 kDa
50 kDa
37 kDa

Figure 4A

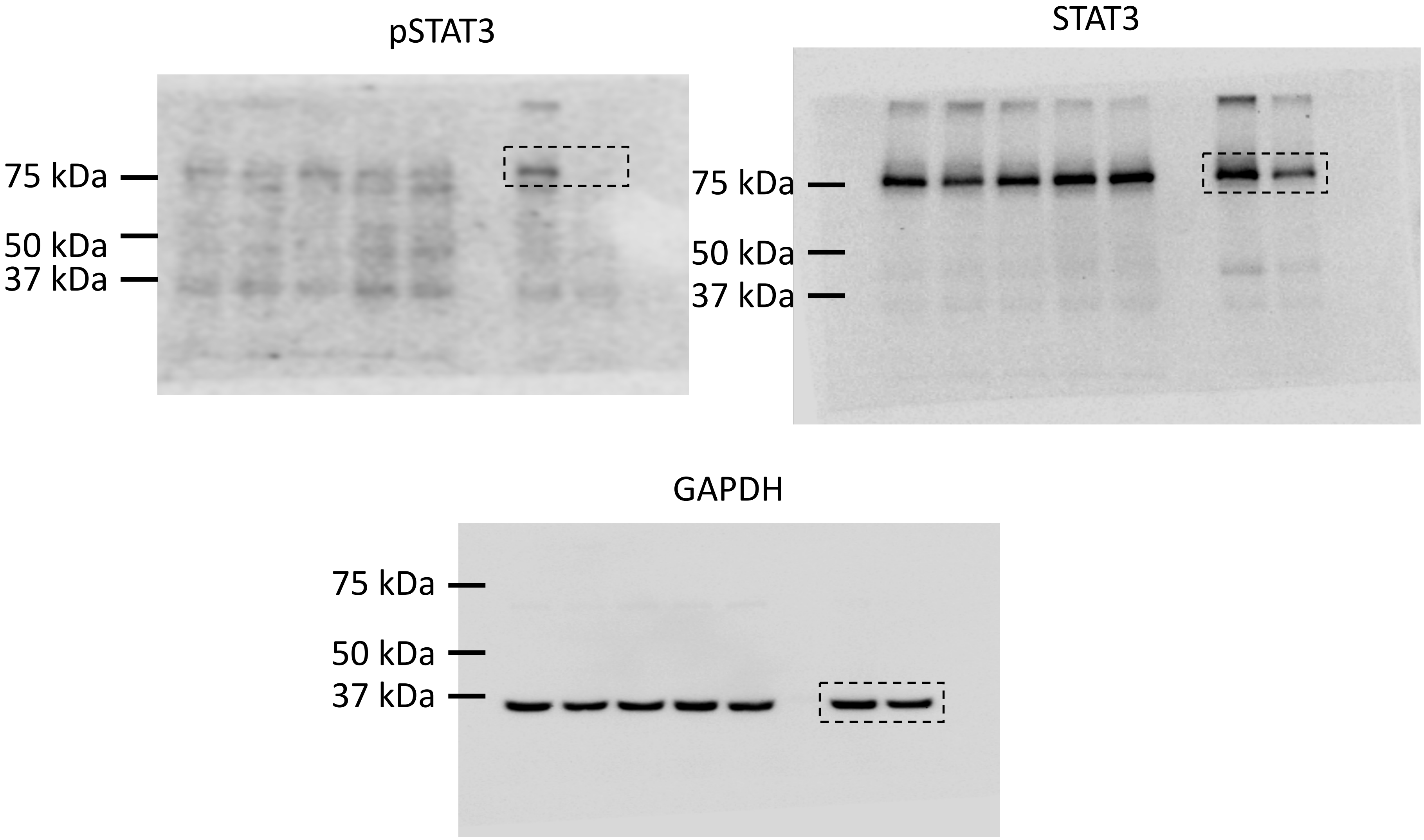


Figure 4D

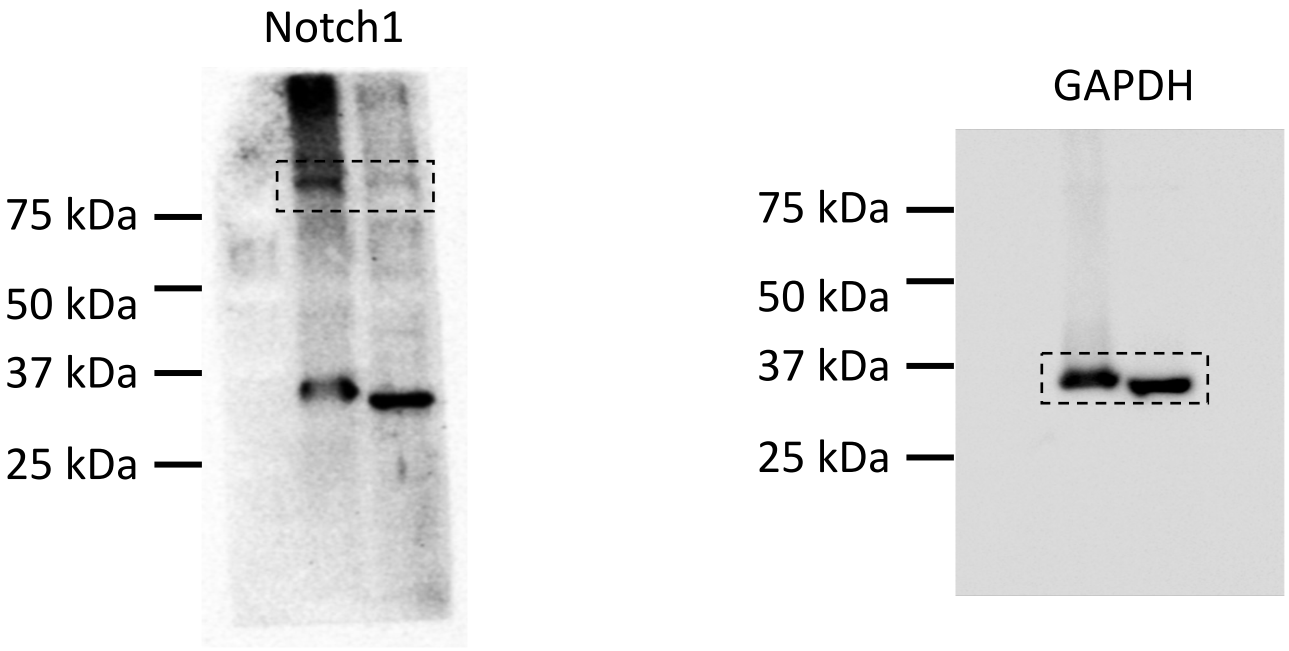
Notch1
75 kDa
50 kDa
37 kDa
25 kDa
GAPDH
75 kDa
50 kDa
37 kDa
25 kDa

Figure 4D

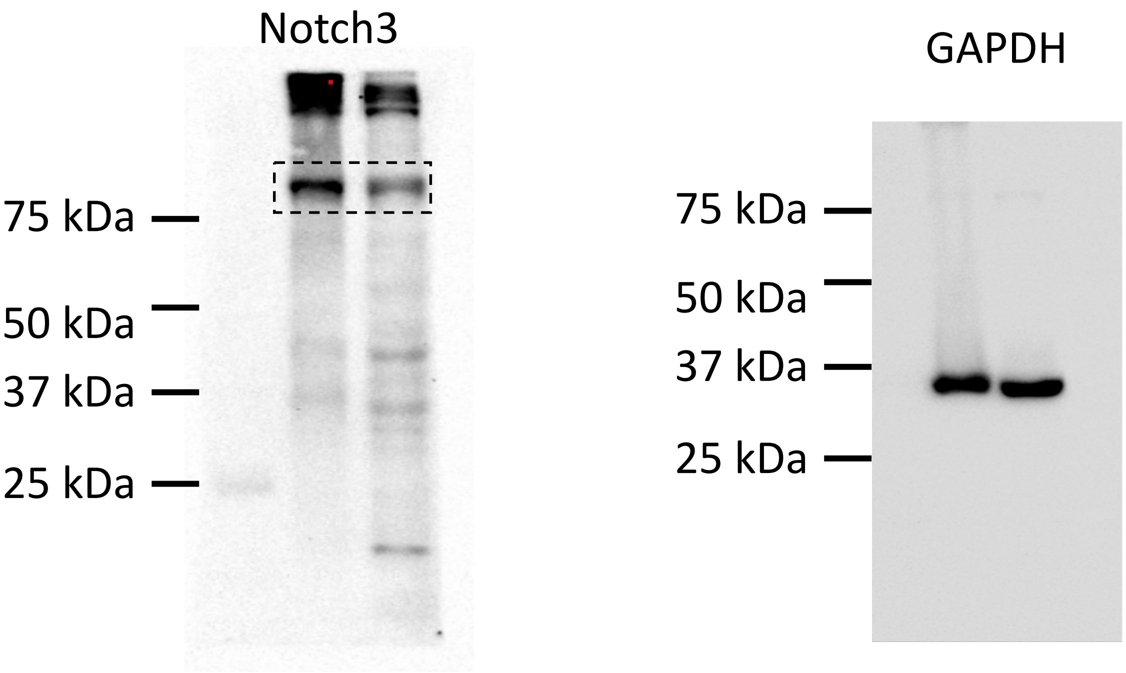
Notch3
75 kDa
50 kDa
37 kDa
25 kDa
GAPDH
75 kDa
50 kDa
37 kDa
25 kDa

Figure 4D

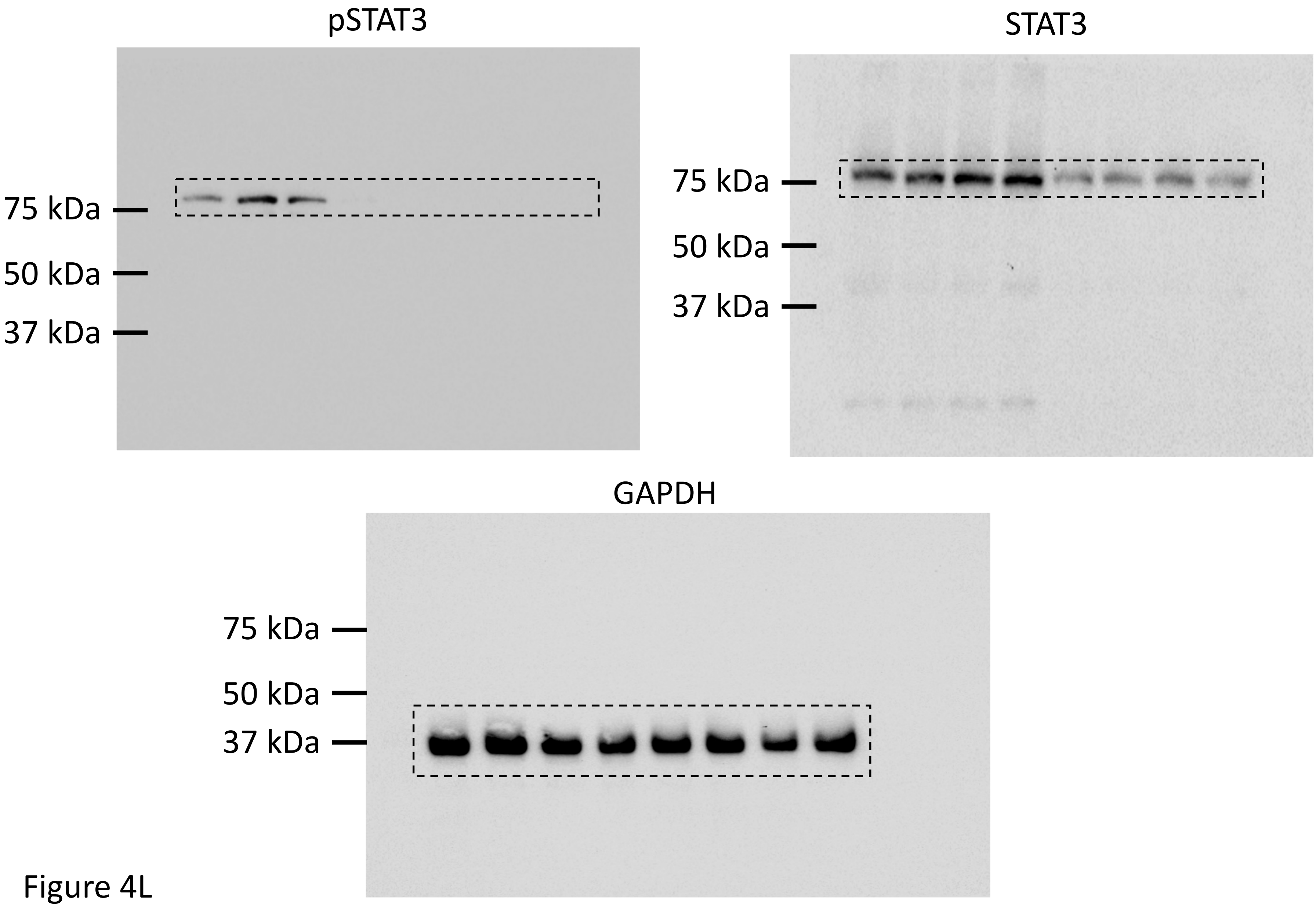


Figure 4L

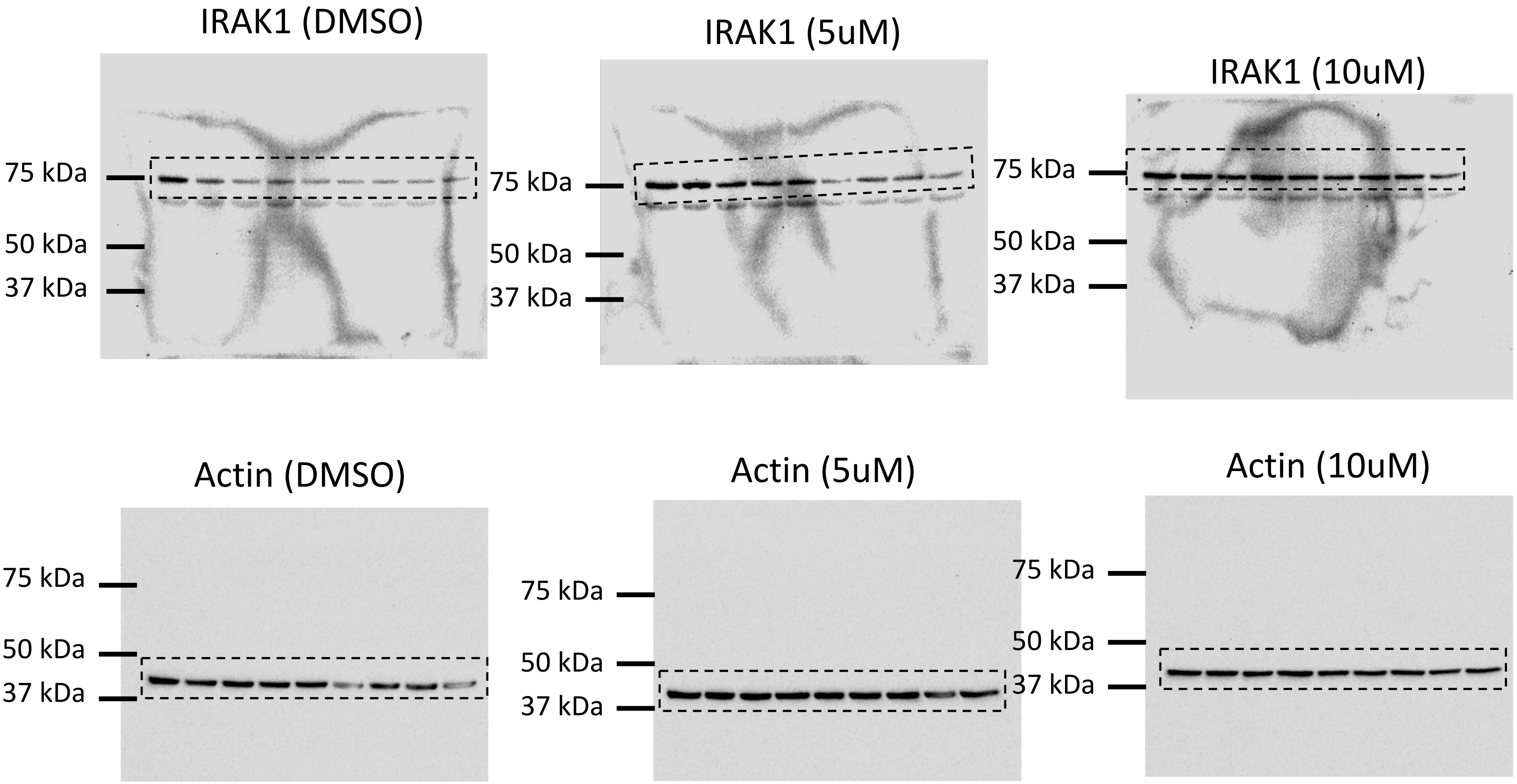
IRAK1 (DMSO)
IRAK1 (5uM)
IRAK1 (10uM)
Actin (DMSO)
Actin (5uM)
Actin (10uM)
75 kDa
50 kDa
37 kDa

Figure 5D

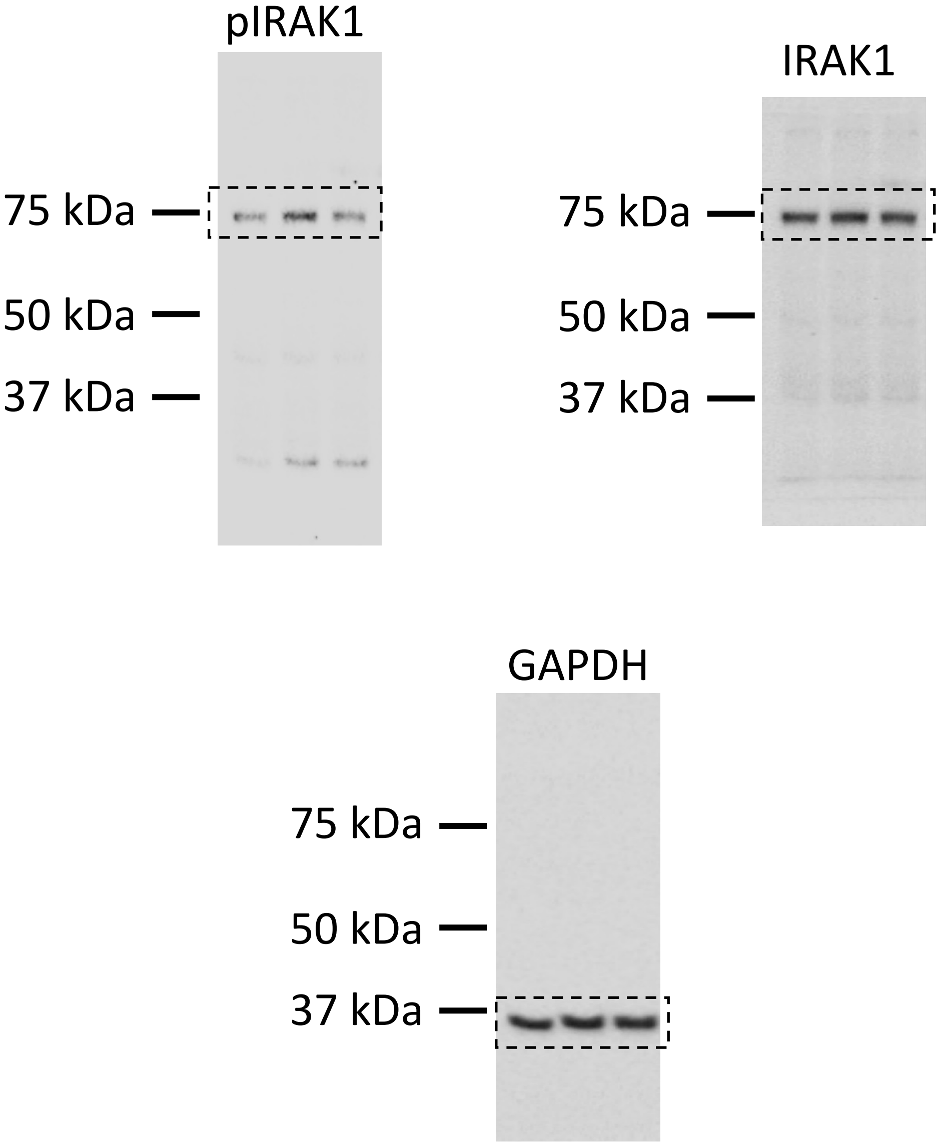
pIRAK1
75 kDa
50 kDa
37 kDa
IRAK1
75 kDa
50 kDa
37 kDa
GAPDH
75 kDa
50 kDa
37 kDa

Figure 5F

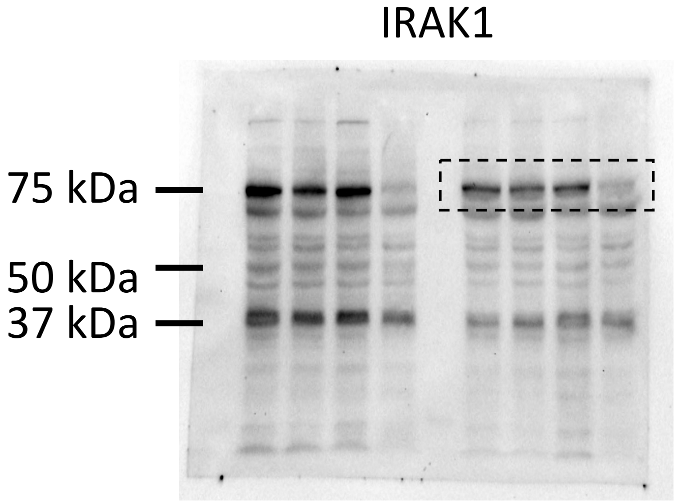


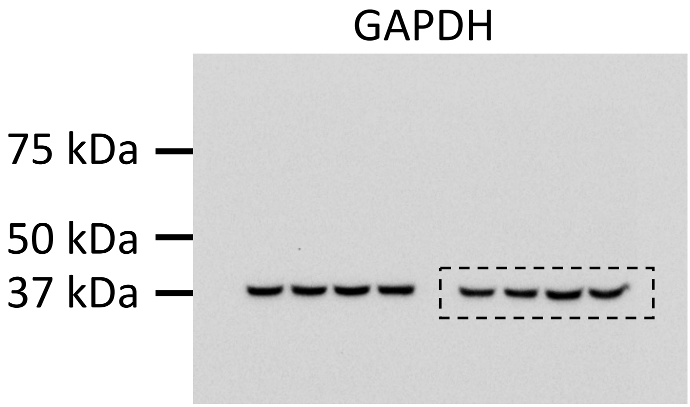


Figure 6G-A1847

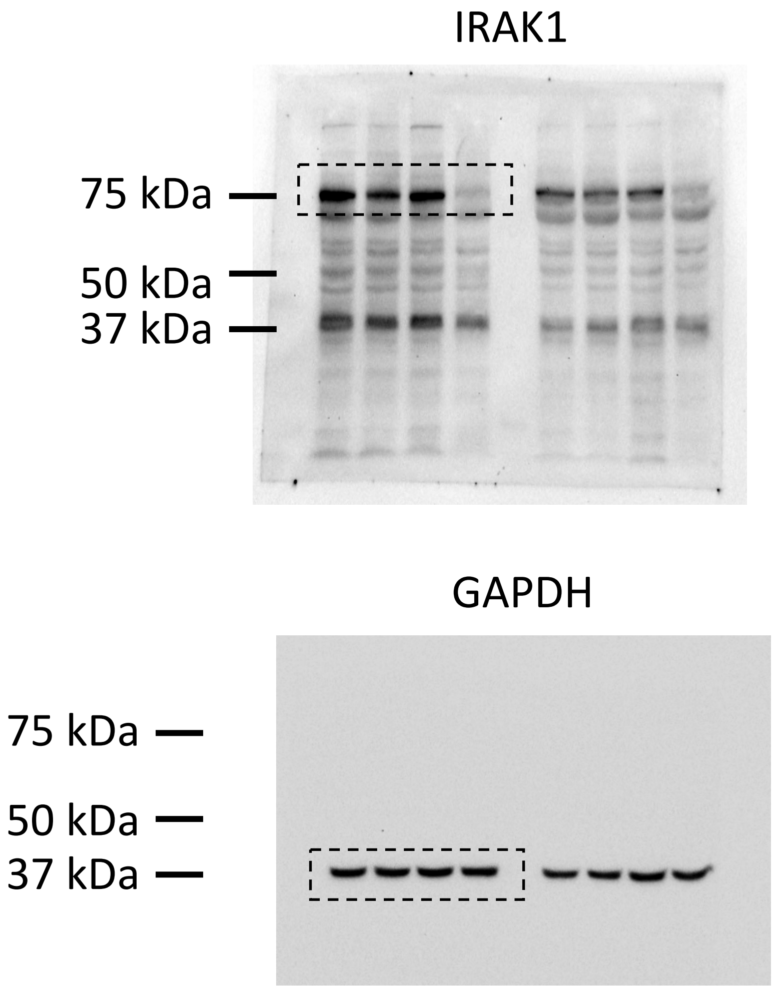


Figure 6G-OVCAR8

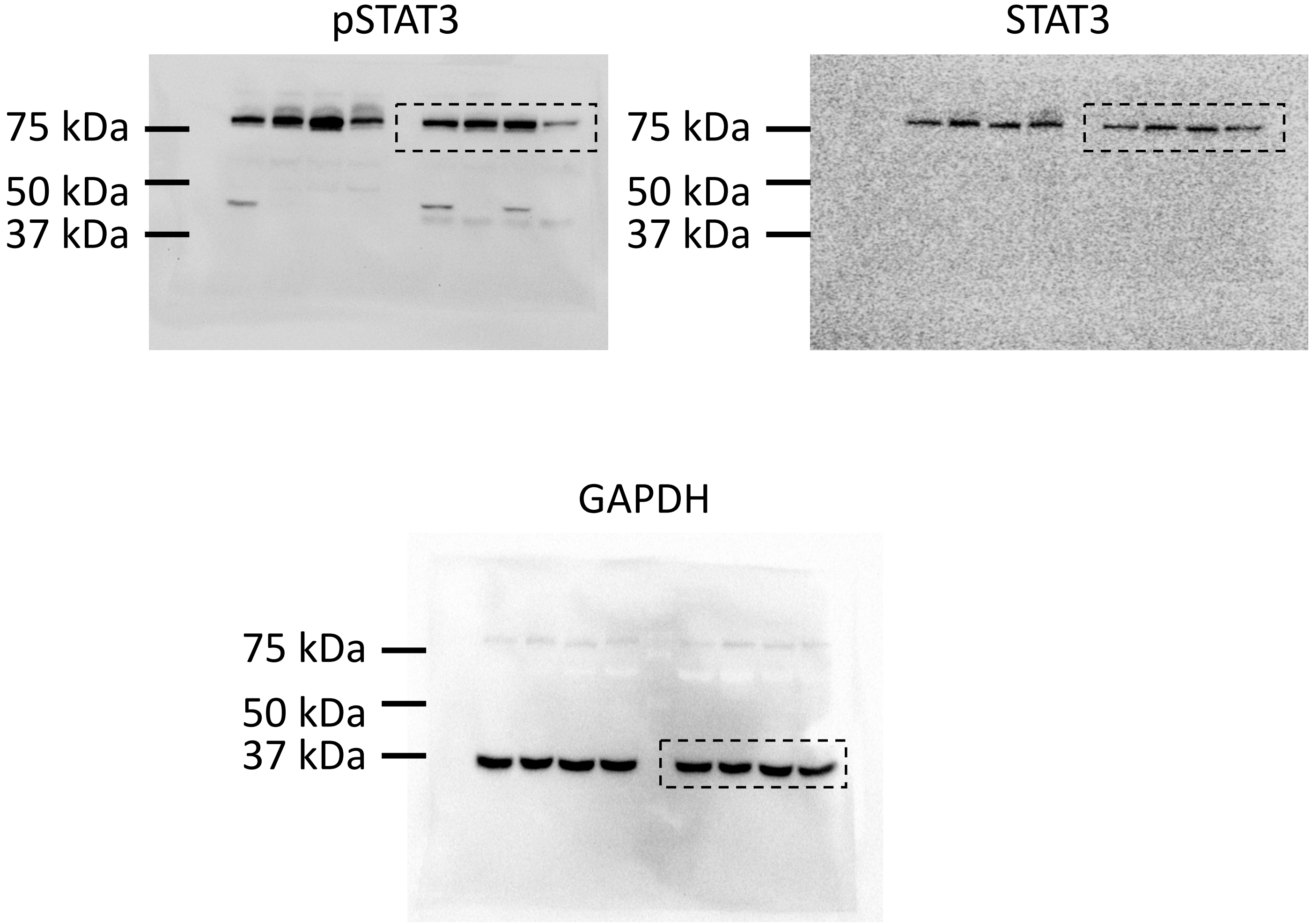


Figure 6G-A1847

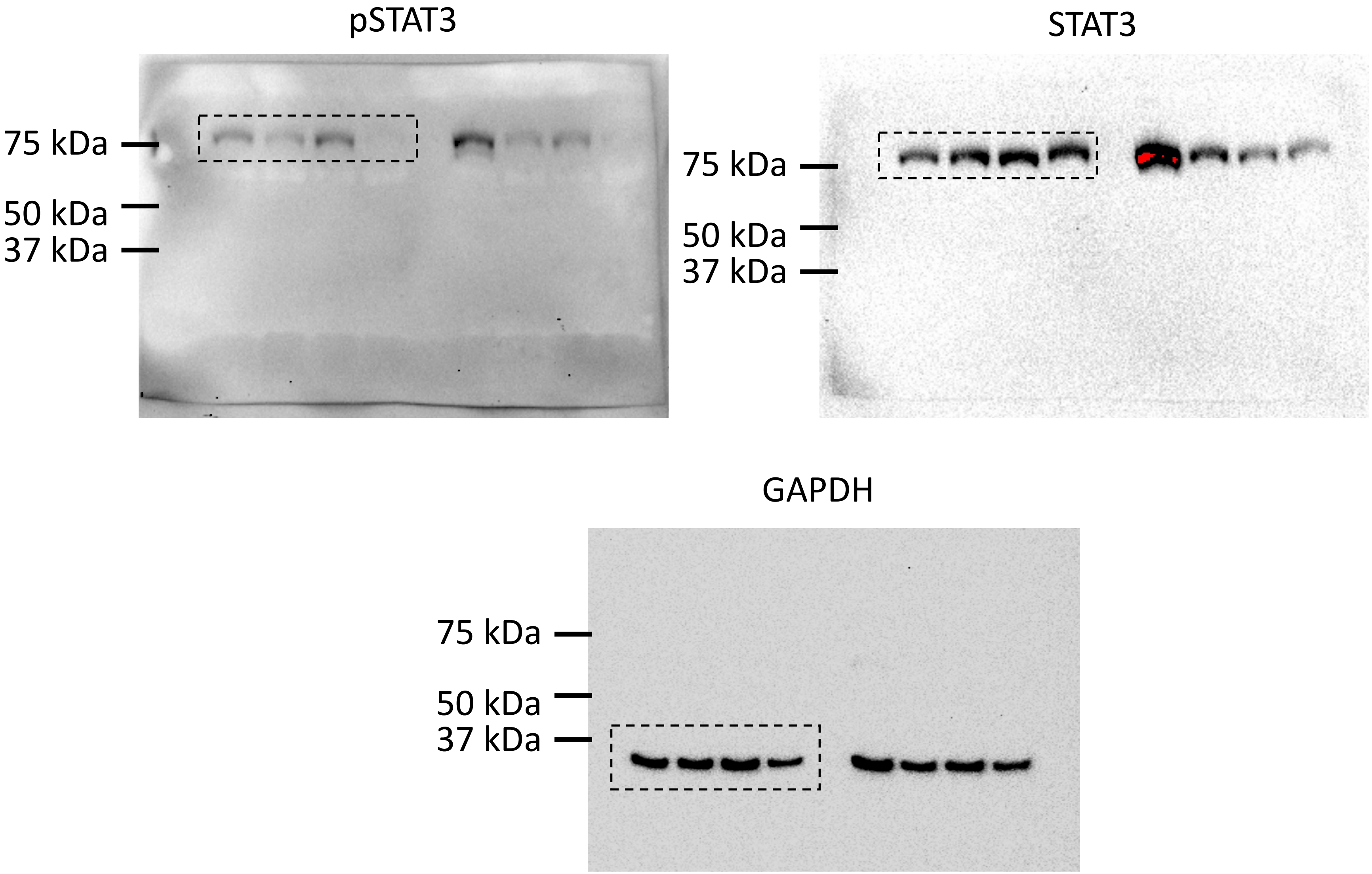


Figure 6G-OVCAR8

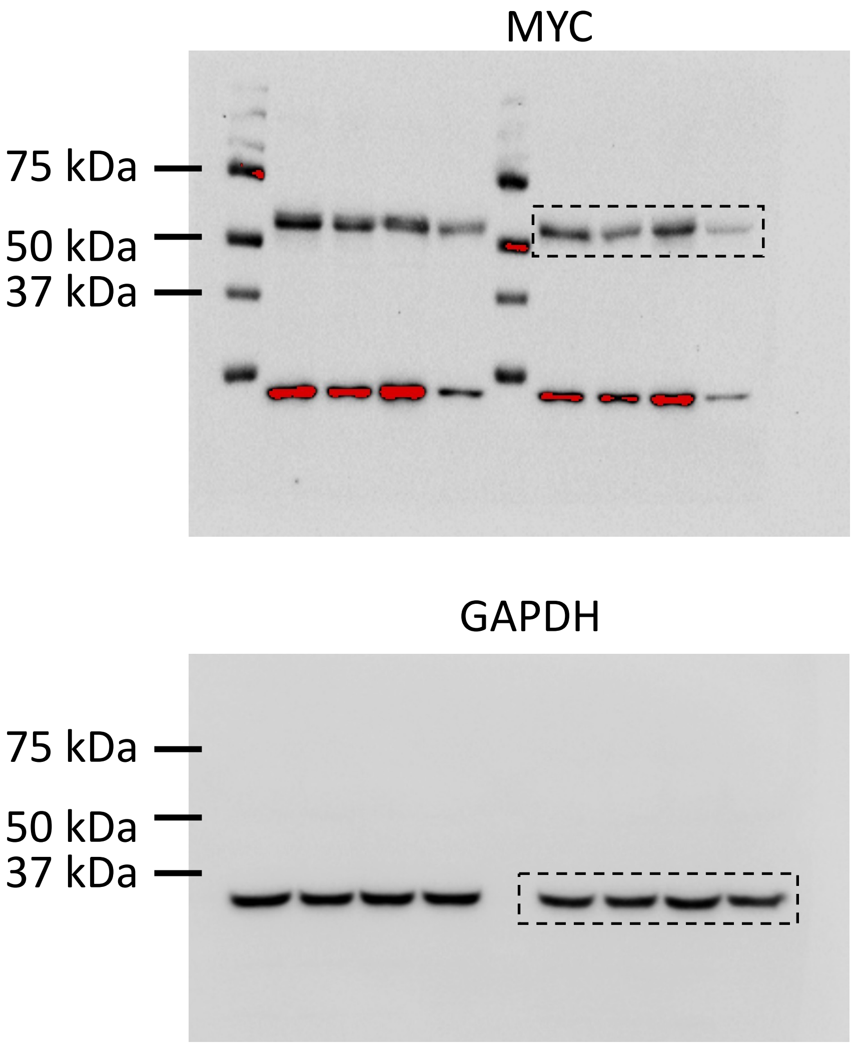


Figure 6G-A1847

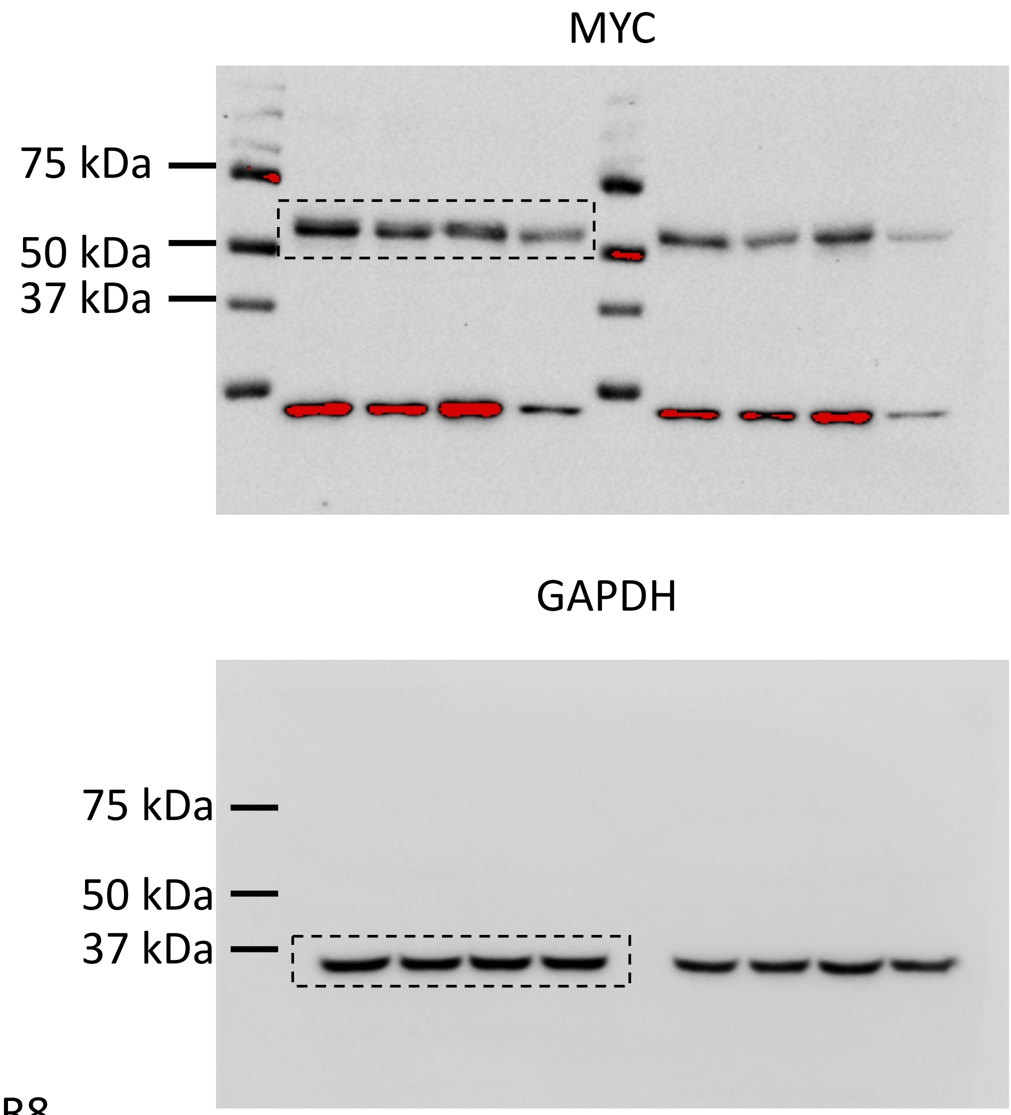


Figure 6G-OVCAR8

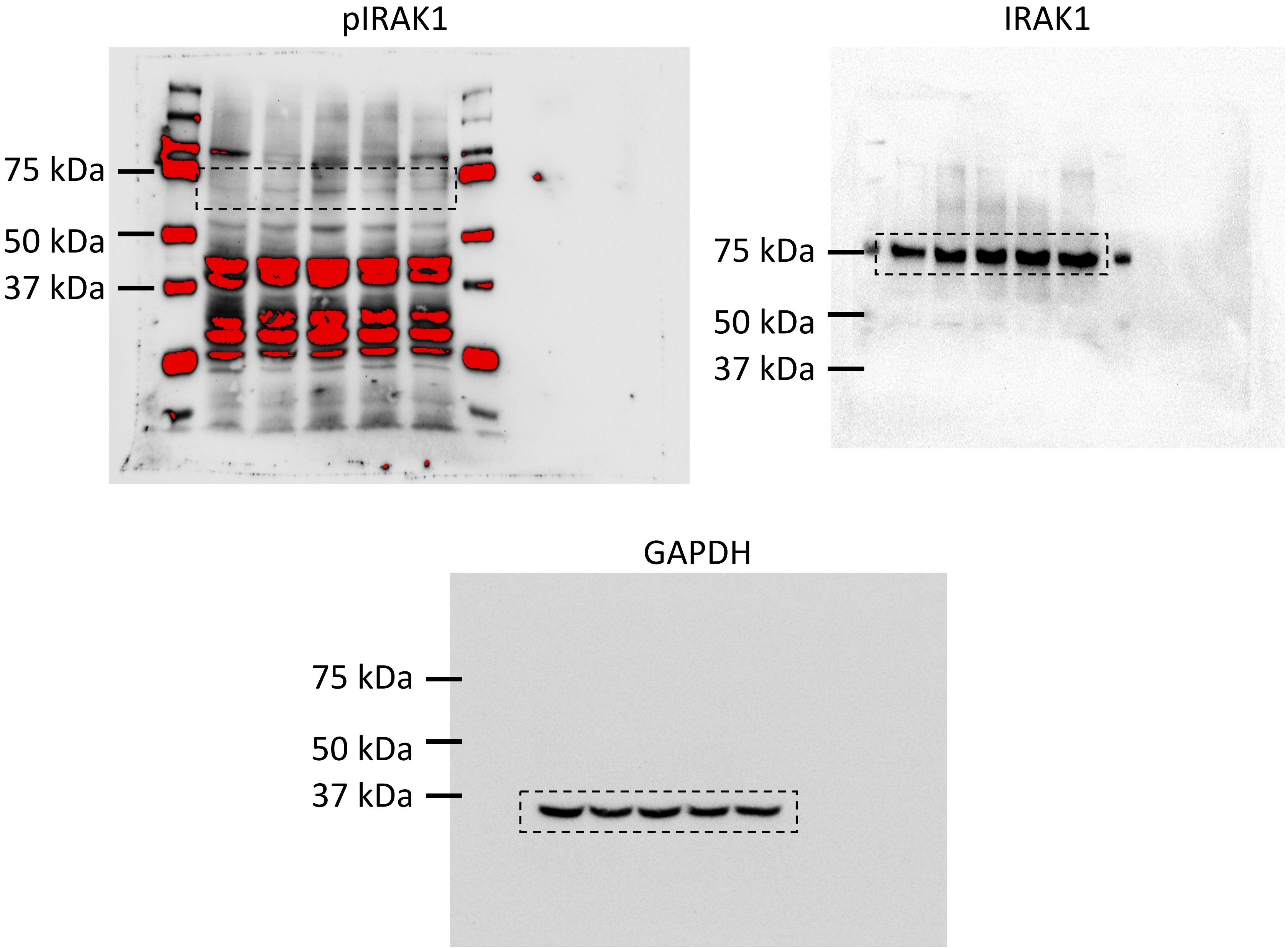


Supplementary Figure 3A-A1847

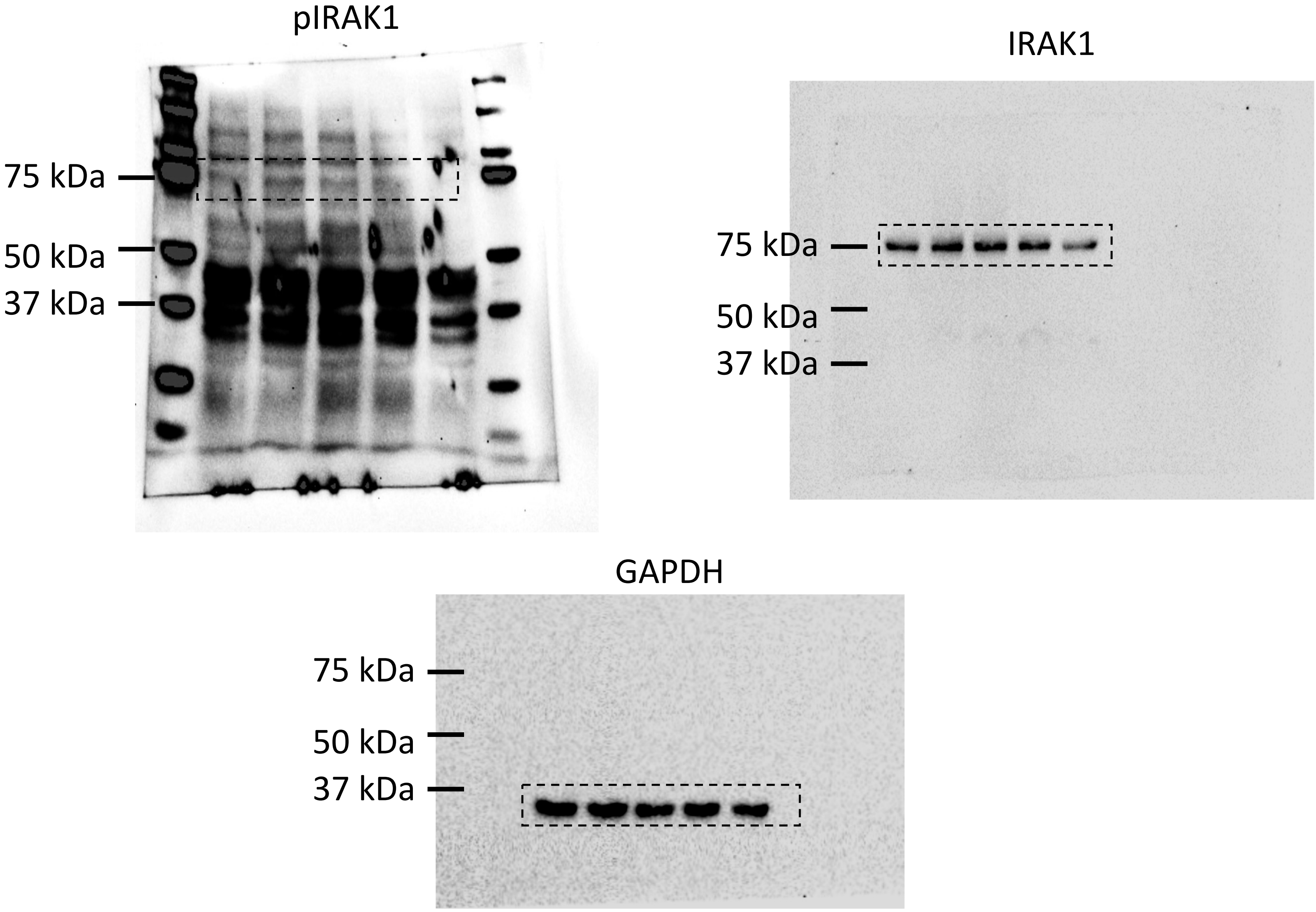


Supplementary Figure 3A-OVCAR8

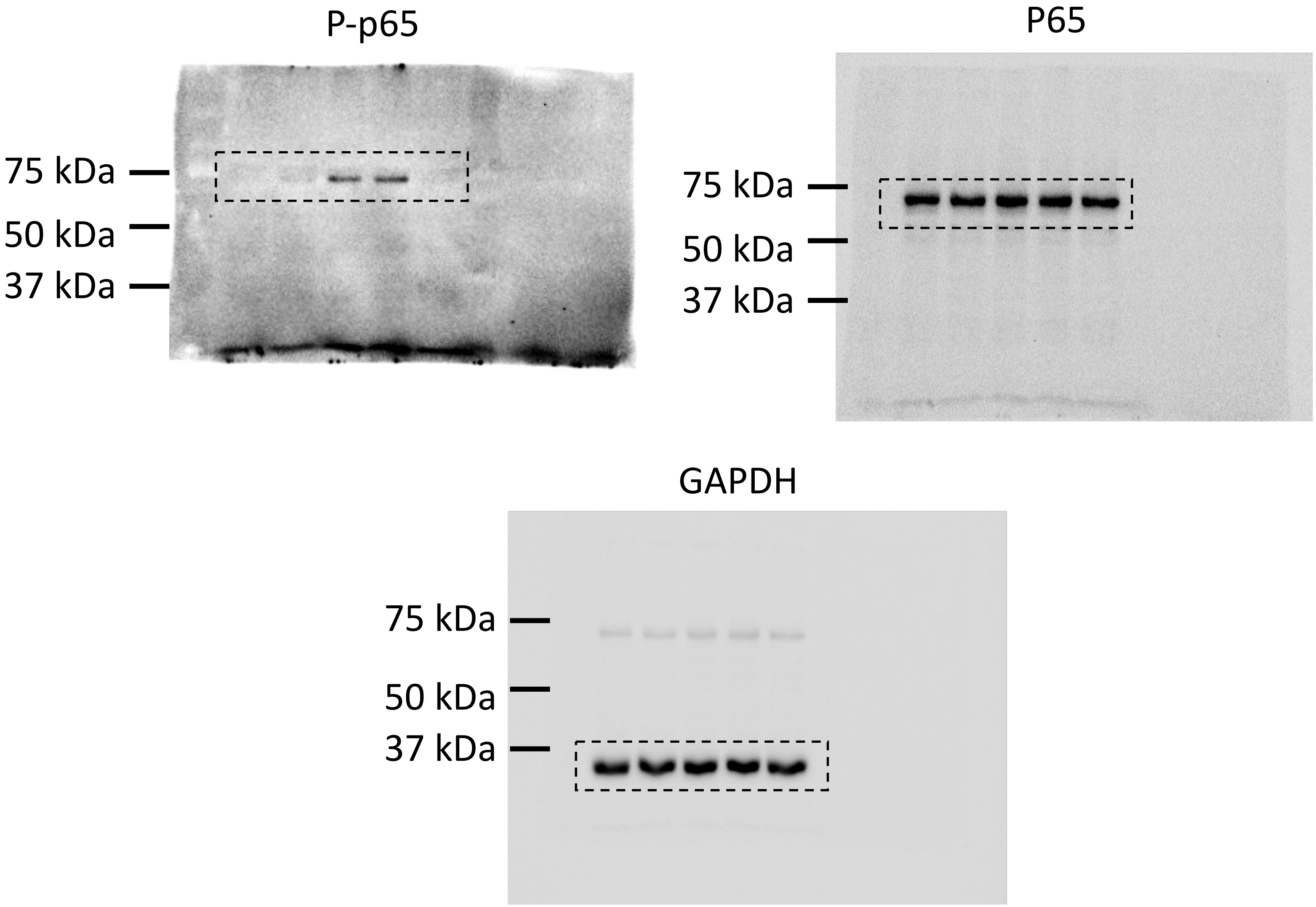


Supplementary Figure 3A-A1847

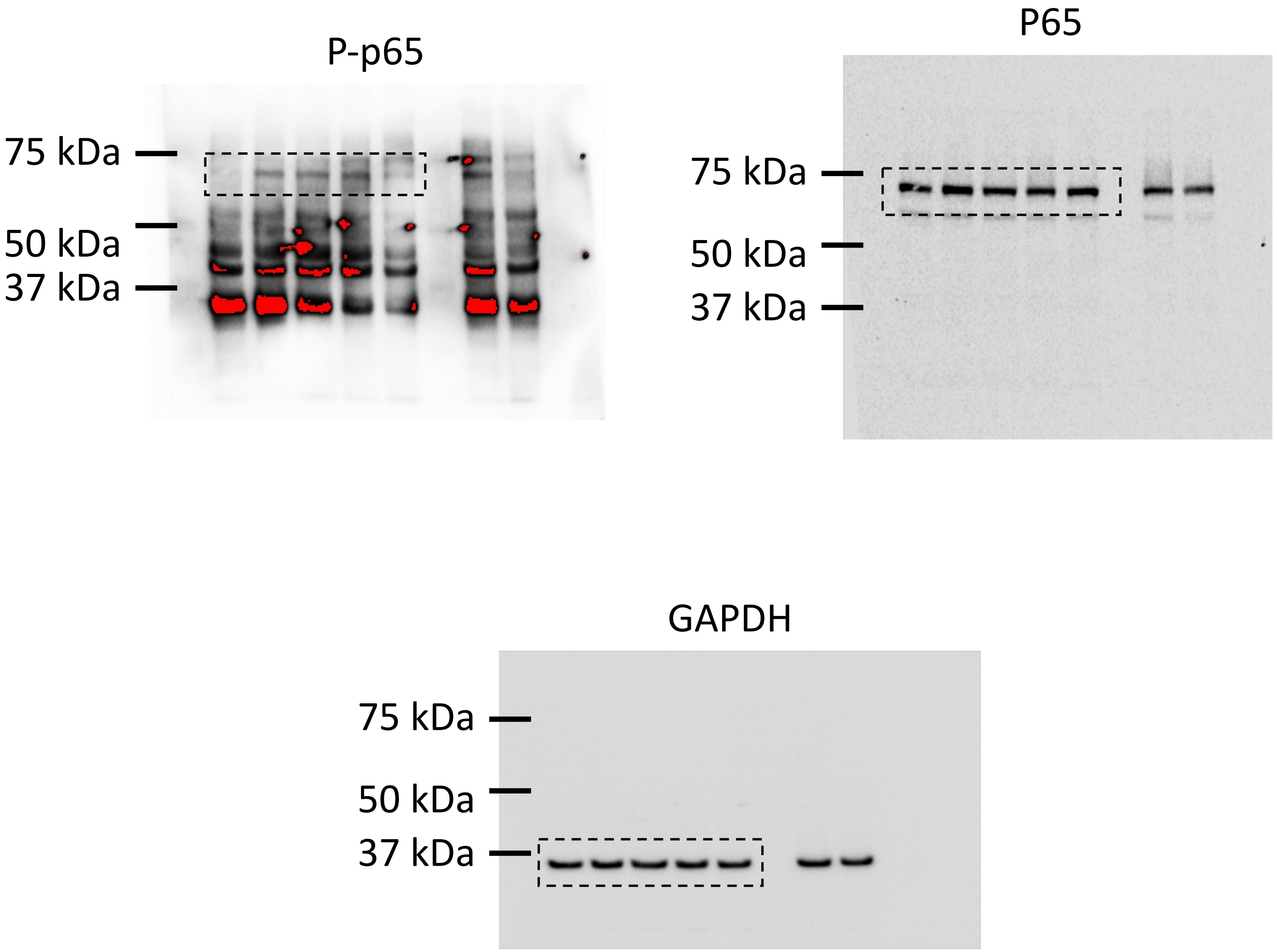


Supplementary Figure 3A-OVCAR8

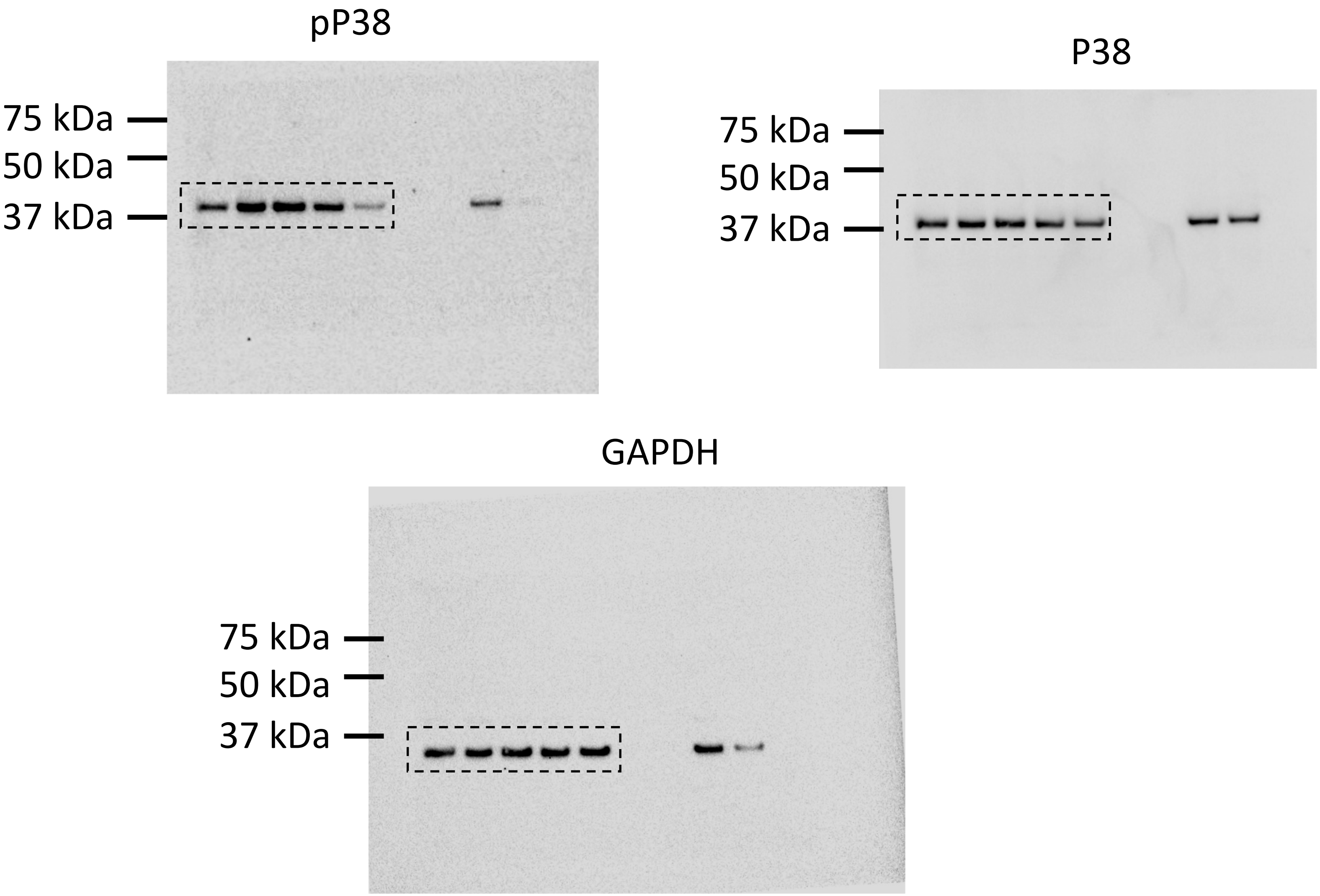


Supplementary Figure 3A-A1847

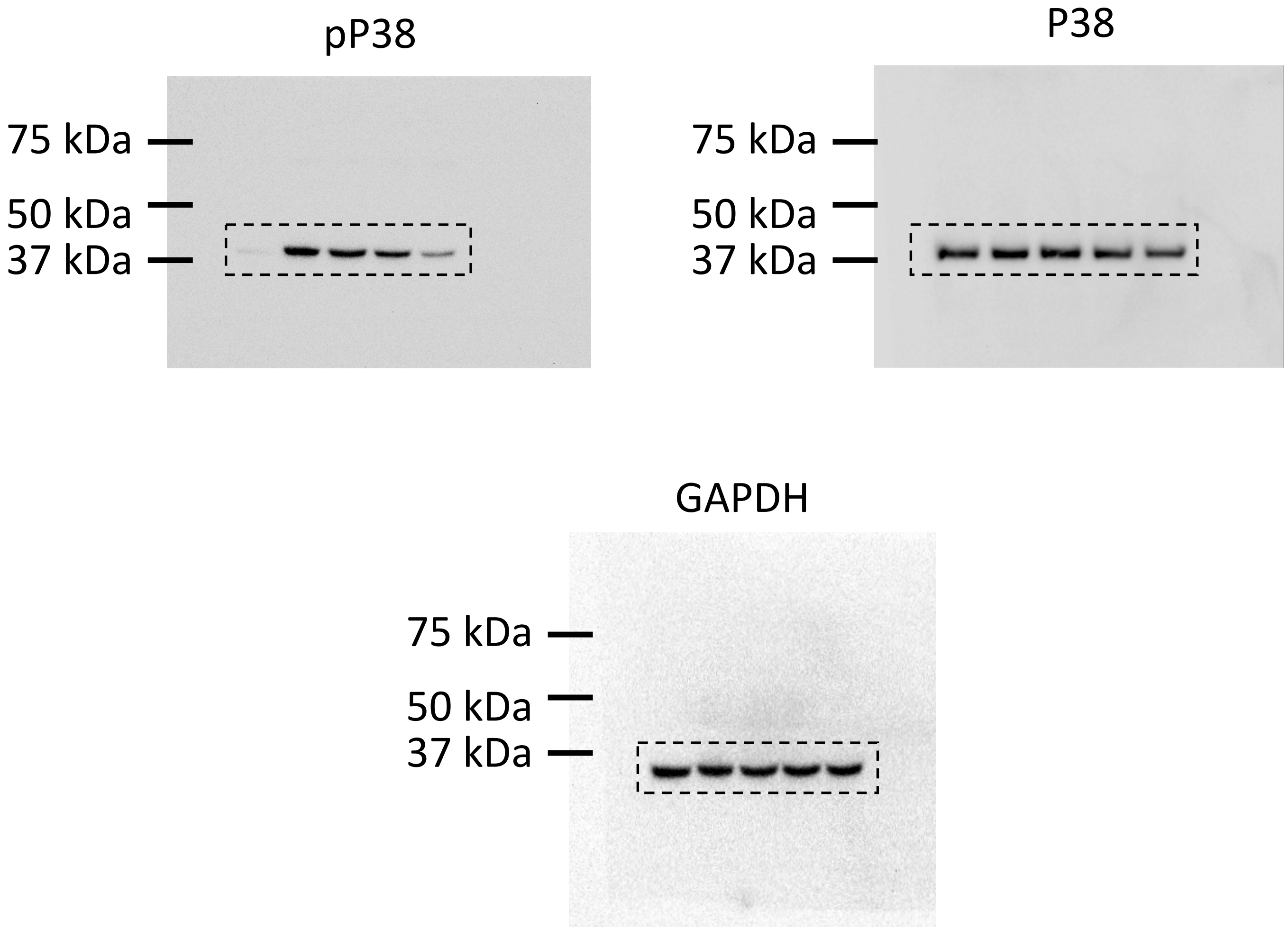


Supplementary Figure 3A-OVCAR8
