# Supplementary material for: Selective targeting of IRAK1 attenuates low molecular weight hyaluronic acid-induced stemness and non-canonical STAT3 activation in epithelial ovarian cancer
Source: Cell Death Dis. 2024 May 25;15(5):362. doi: 10.1038/s41419-024-06717-3 (PMC11127949; doi:10.1038/s41419-024-06717-3)

**Supplementary Figure 1**. (A) Quantification of TIR signaling gene expression from NCBI GEO Database (GDS1381) comparing expression between carboplatin resistant and sensitive patients. (B) Quantification of TIR signaling gene expression from NCBI GEO Database (GDS5227) comparing 3-D culture effect on fallopian tube secretory epithelial cells. (C) Quantification of stemness and multi-drug resistant gene expression from RNA-sequencing in C30 cells compared to A2780 cells grown in 2-D cultures. (D) Quantification of stemness and multi-drug resistant gene expression from RNA-seq of A2780 cells grown in 3-D cultures compared to 2-D. (E) Quantification of IPSC stemness gene expression from NCBI GEO Database (GDS1381) comparing expression between carboplatin resistant and sensitive patients. (F) Quantification of multi-drug resistant gene expression from NCBI GEO Database (GDS1381). (G) Quantification of IPSC stemness gene expression from NCBI GEO Database (GDS5227) comparing 3-D culture effect on fallopian tube secretory epithelial cells. (H) Quantification of multi-drug resistant gene expression from NCBI GEO Database (GDS5227). Data are presented as mean showing individual technical replicates ± SEM from 3 independent patients. * $p<0.05$, ** $p<0.01$, *** $p<0.001$, **** $P<0.0001$, NS=not significant.

**Supplementary Figure 2.** (A) Quantification of copy number alterations in TIR signaling receptors from 3 independent cBioPortal ovarian serous adenocarcinoma datasets. 1: Firehose Legacy 2: PanCancer Atlas 3: Nature 2011. (B) Quantification of copy number alterations in TIR signaling mediators as in (A). (C) Quantification of copy number alterations in TIR transcription factors as in (A). (D) Oncoprint of genetic alterations comparing IRAK1 and genes related to *BRCA* and DNA damage repair from cBioPortal ovarian serous cystadenocarcinoma Firehose Legacy dataset. (E) Graphical representation of correlations comparing IRAK1 mRNA and BRCA1 or BRCA2 expression from cBioPortal ovarian serous cystadenocarcinoma Firehose Legacy dataset. (F) Quantification of IRAK1 mRNA expression from NCBI GEO Database (GDS3592) comparing ovarian normal surface epithelia and ovarian cancer epithelial cells. (G) Quantification of IRAK1 mRNA expression from NCBI GEO Database (GDS1381) comparing carboplatin resistance in patients. (H) Quantification of IRAK1 mRNA expression from NCBI GEO Database (GDS3754) comparing A2780 epithelial ovarian cancer cell line with acquired platinum resistance. Data are presented as mean ± SEM. Statistical significance was calculated by 2-way ANOVA ** $p<0.01$, *** $p<0.001$.

**Supplementary Figure 3.** (A) Western blot time course in A1847 and OVCAR8 cells following stimulation with or without IL1β (10 ng/mL) for phosphorylated and total IRAK1, p65, and p38. (B) Quantification of CD44 mRNA from TCGA ovarian cancer dataset in normal tissue (NT), primary tumor (PT), and recurrent tumor (RT). Data is represented as a bloxplot, min to max, showing all data points. (C) Correlation of CD44 mRNA versus IRAK1 mRNA, represented as log2 FPKM from GDC TCGA EOC dataset (n=379). (D) Graphical correlation of (C). Red represents high expression. Blue represents low expression.

**Supplementary Figure 4**. (A) Hexosaminidase assay of A2780, C30, and SKOV3 cells treated with Milipore Sigma IRAK1/4 inhibitor for 72h. (B) Ribbon model of IRAK1 protein, highlighting domain used for *in silico* docking. (C) Schematic of docking workflow for *in silico* screening. (D) Summary of mesenchymal stem cell differentiating compound list from Tocris and binding energies following *in silico* docking screen with IRAK1 within the ligand binding domain. (E) Space-filled models of CW008, troglitazone, H89, and kartogenin binding within the IRAK1 ligand binding domain. Amino acids involved in hydrogen bonding are highlighted.

**Supplementary Figure 5**. (A) Quantification of colony number of A2780 and C30 cells following treatment TCS2210. Data are presented as mean ± SEM from 3 independent experiments. (B) Quantification of colony size as in (A). Data are presented as mean ± SEM from 3 independent experiments. (C) Quantification of number of spheroids of A2780 and C30 cells treated with TCS2210. Data are

presented as mean ± SEM, showing all data points from 3 independent experiments. (D) Hexosaminidase viability assay of A2780 cells treated with TCS2210, cyclodextrin (CD), or TCS2210:CD formulation. (E) Structure of nano-TCS2210 (nano-TCS). (F) Representative electron-micrograph of nano-TCS particles. (G) Quantification of Nano-TCS particle size. (H) Hexosaminidase viability assay of A1847 cells treated with TCS2210 or Nano-TCS.

# Supplementary Figure 1

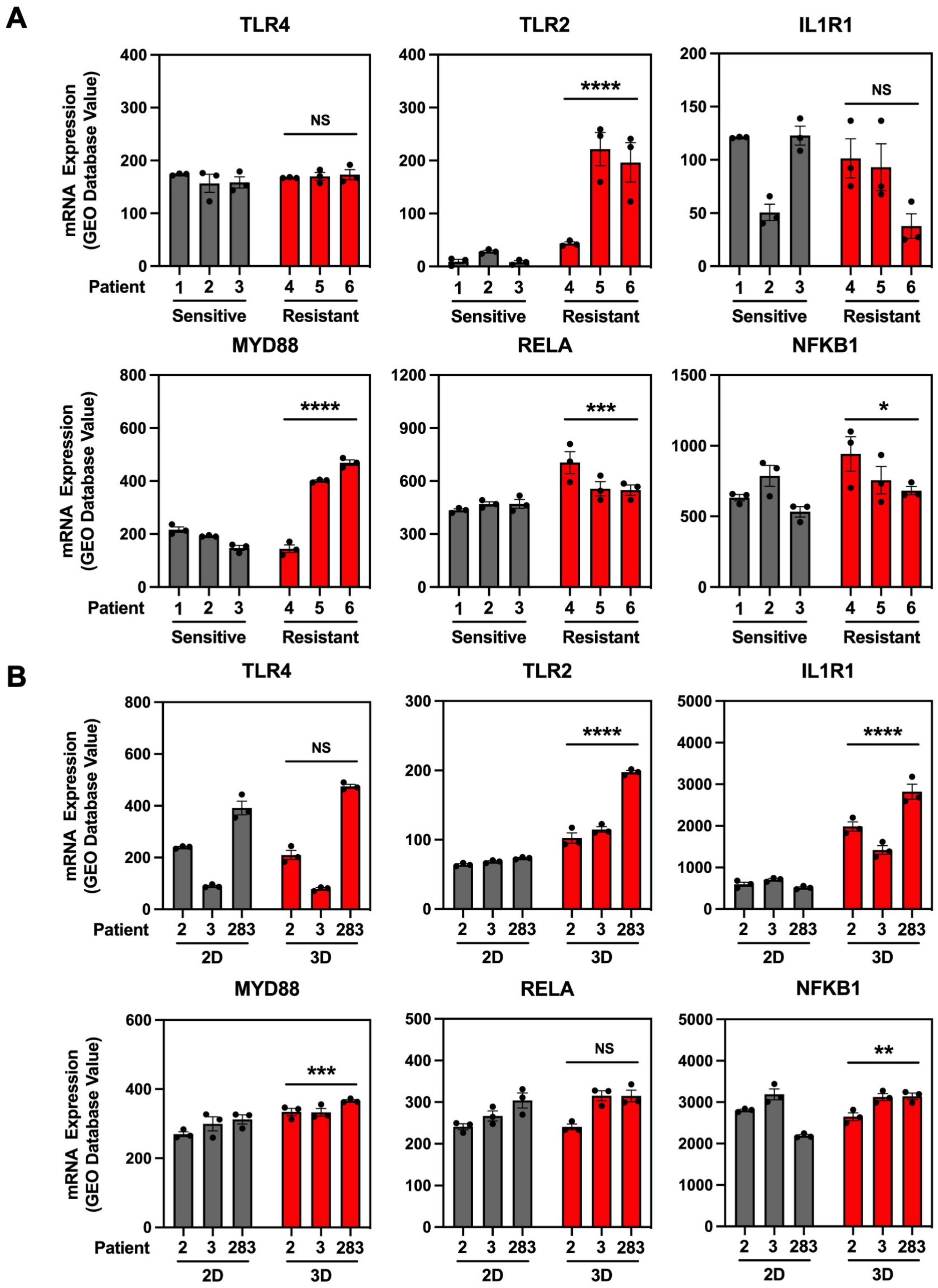

# Supplementary Figure 1

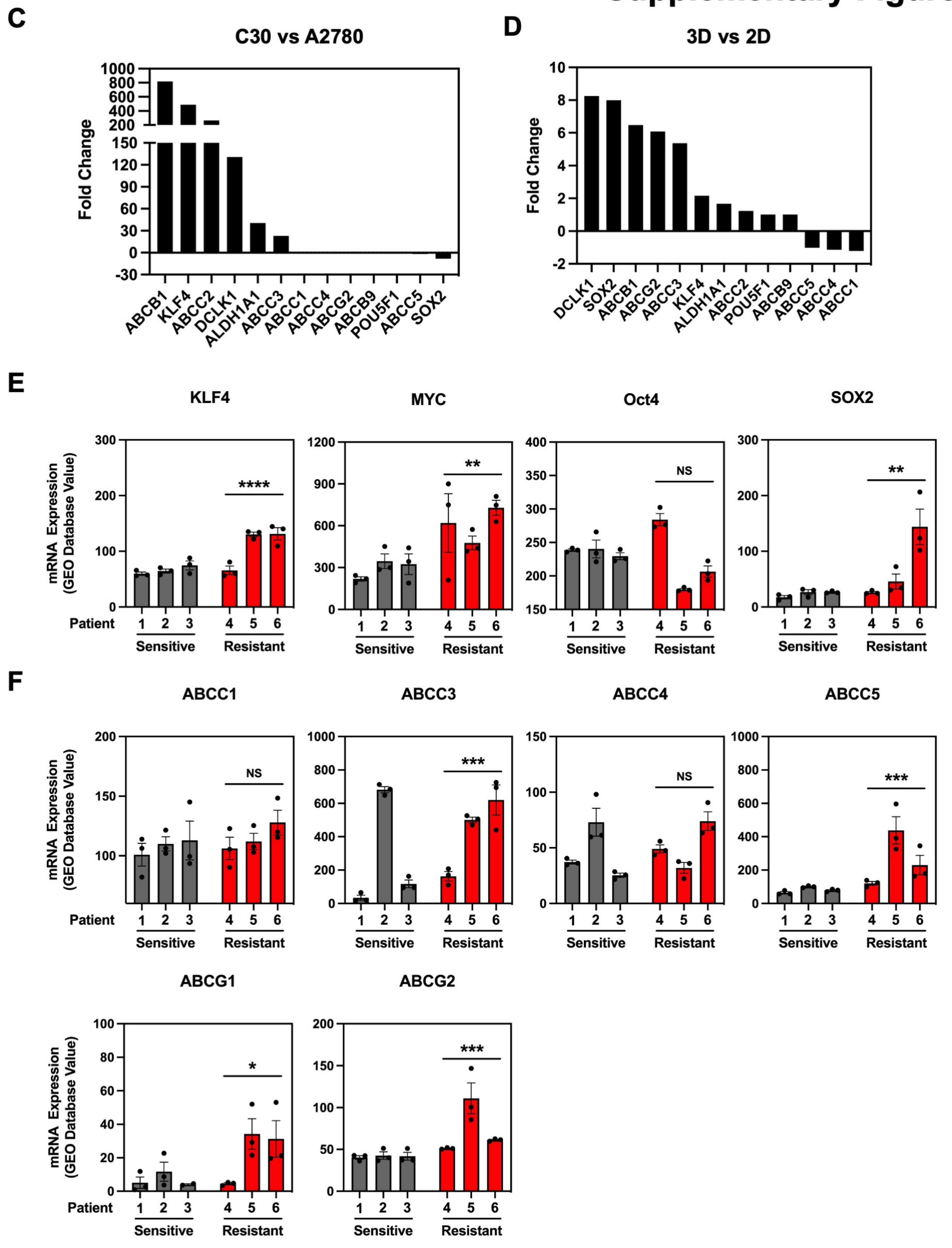

# Supplementary Figure 1

G

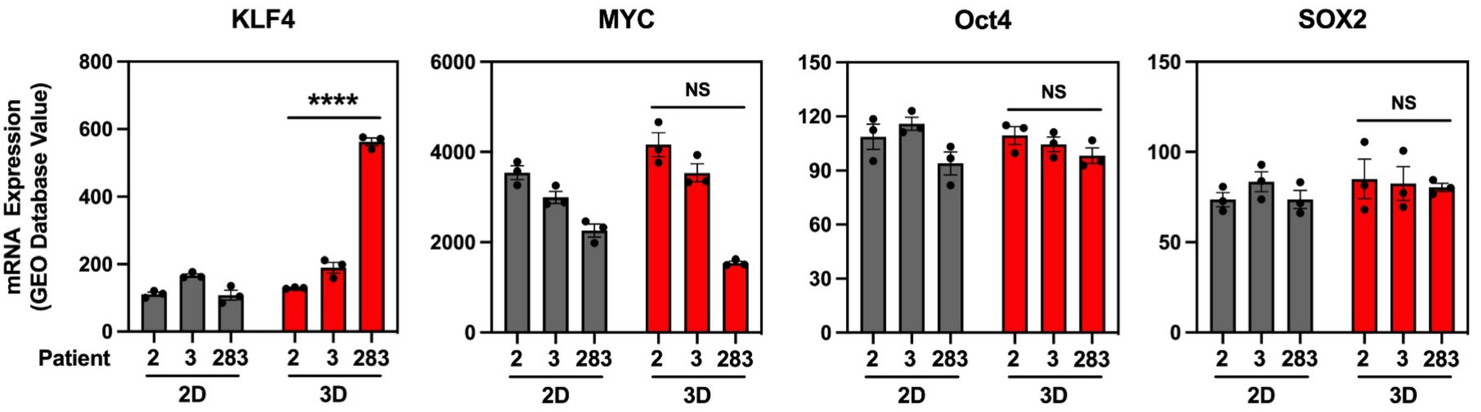


H

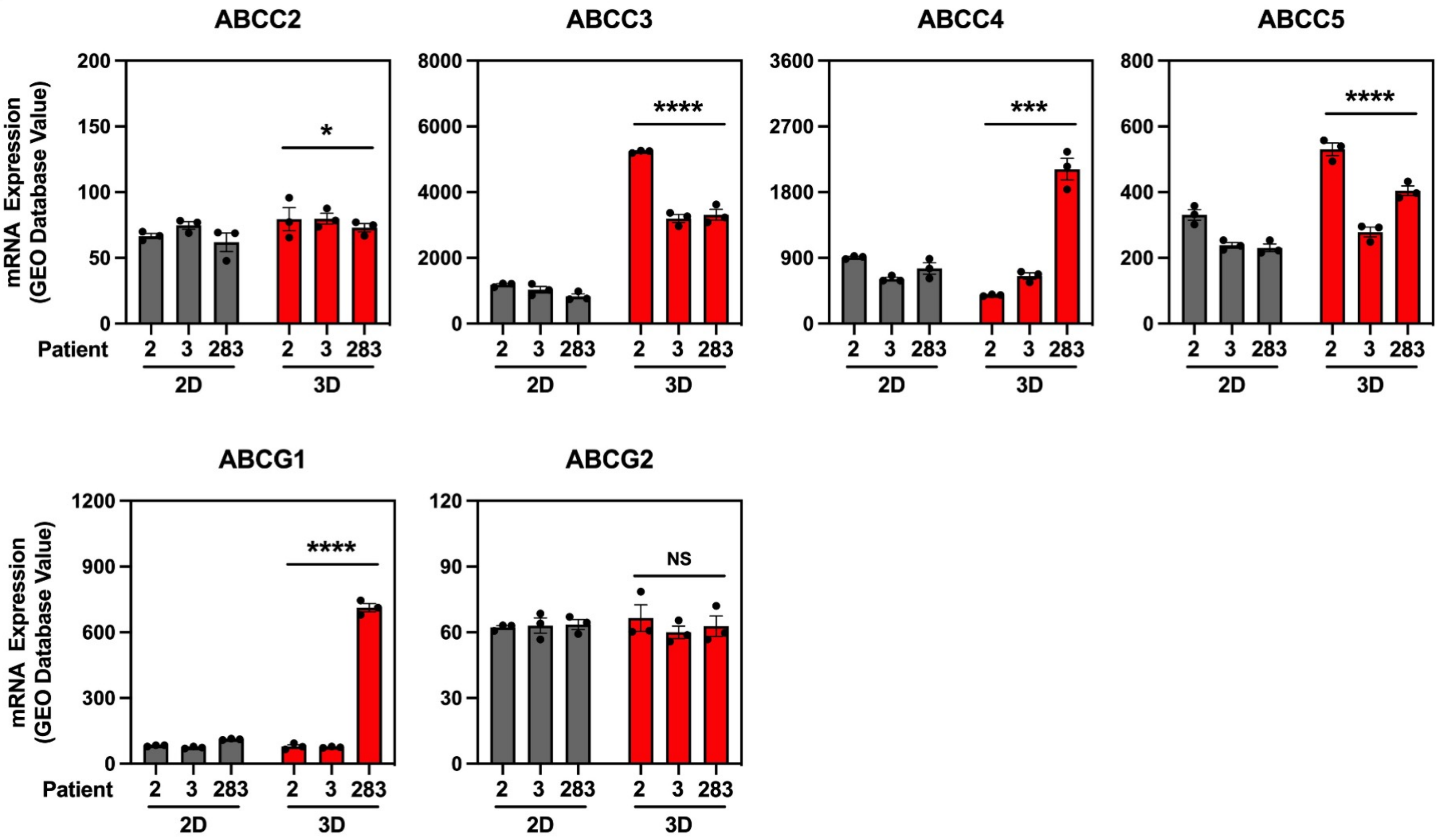

# Supplementary Figure 2

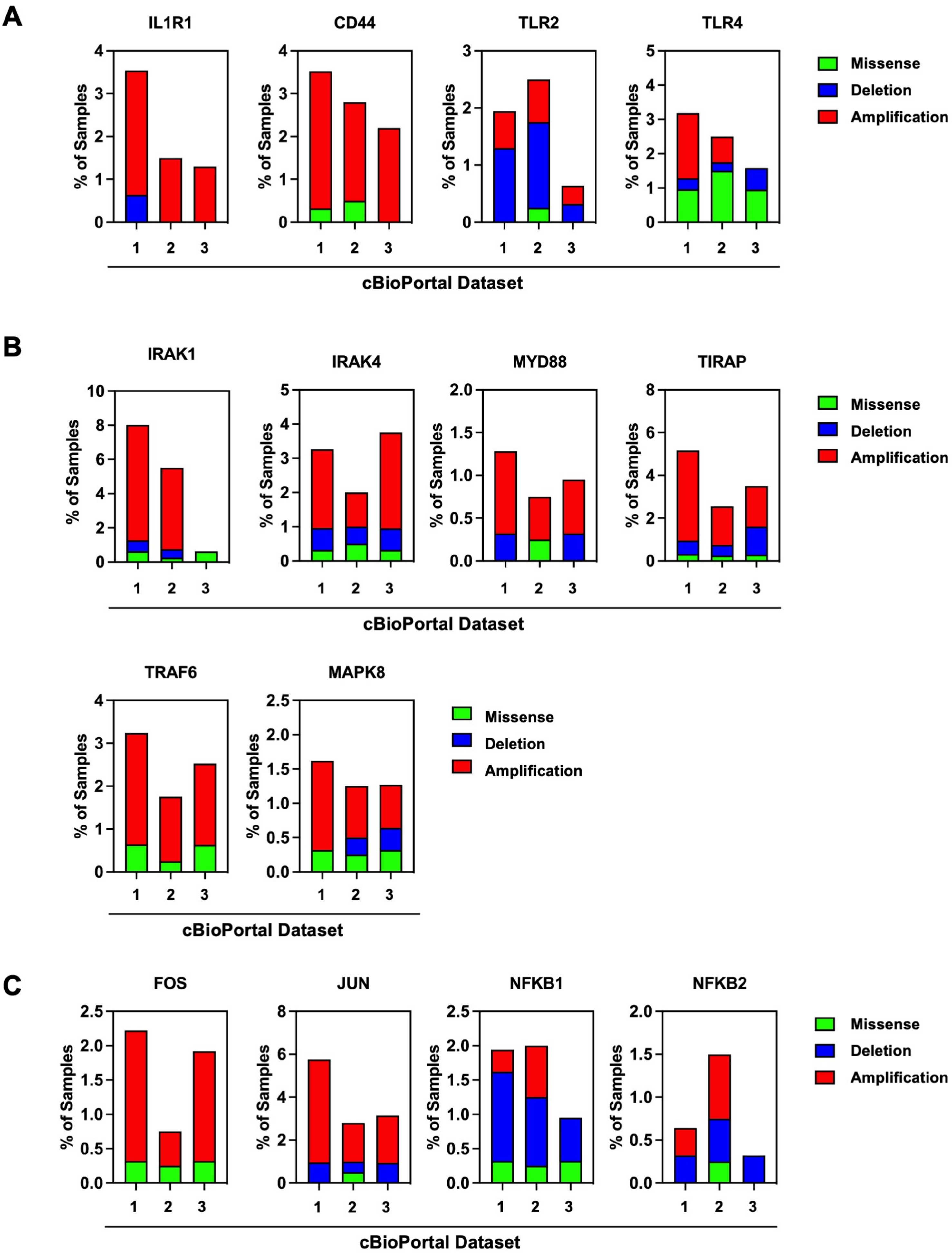

# Supplementary Figure 2

D

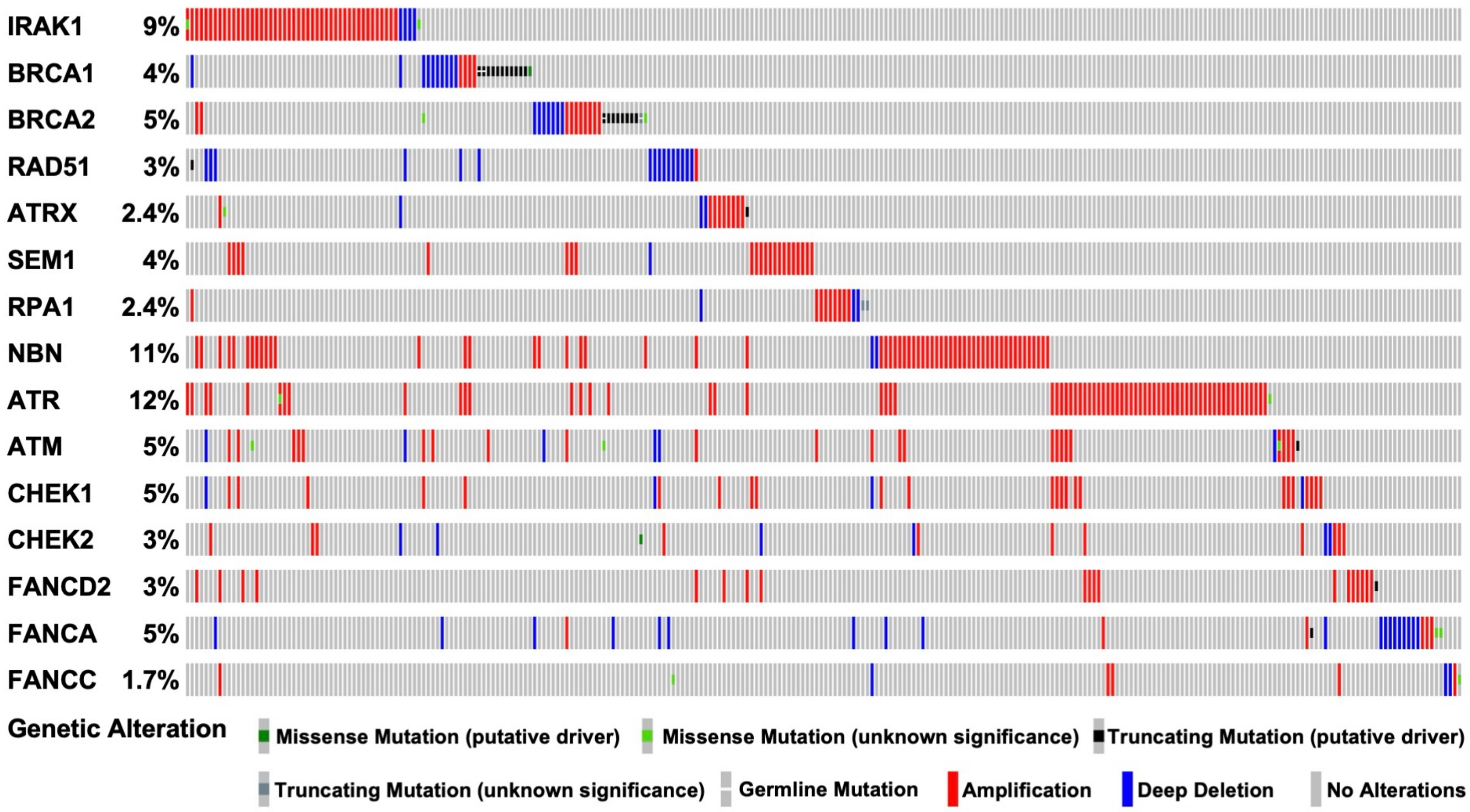


E

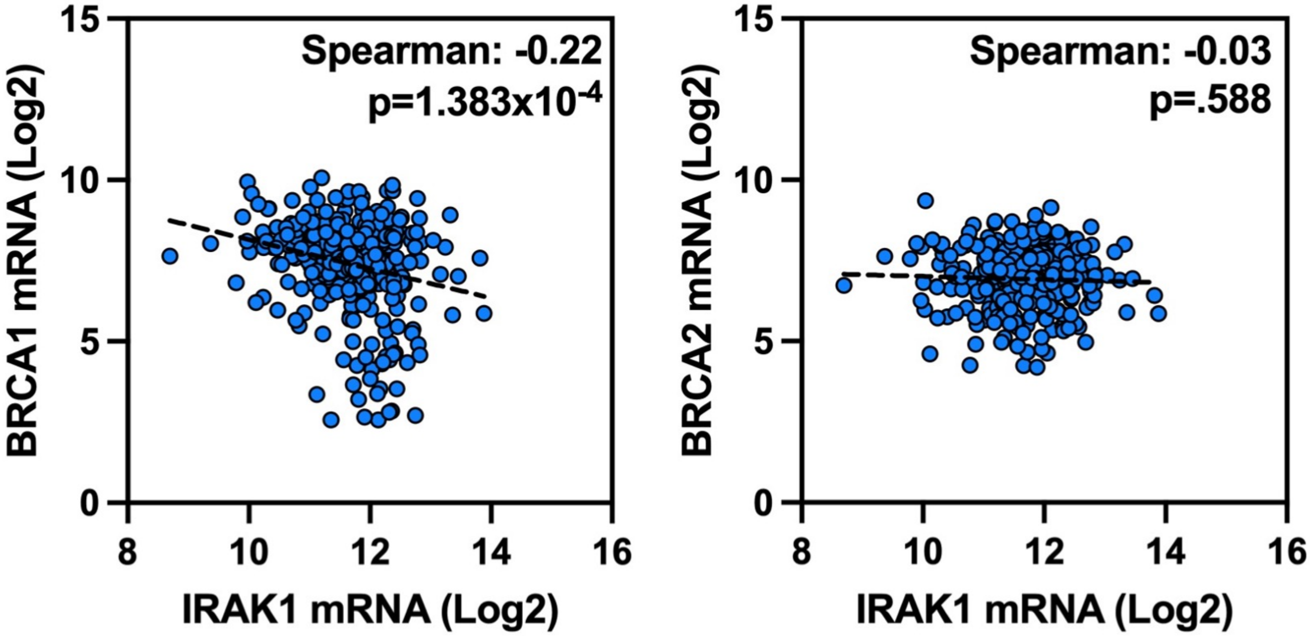

# Supplementary Figure 2

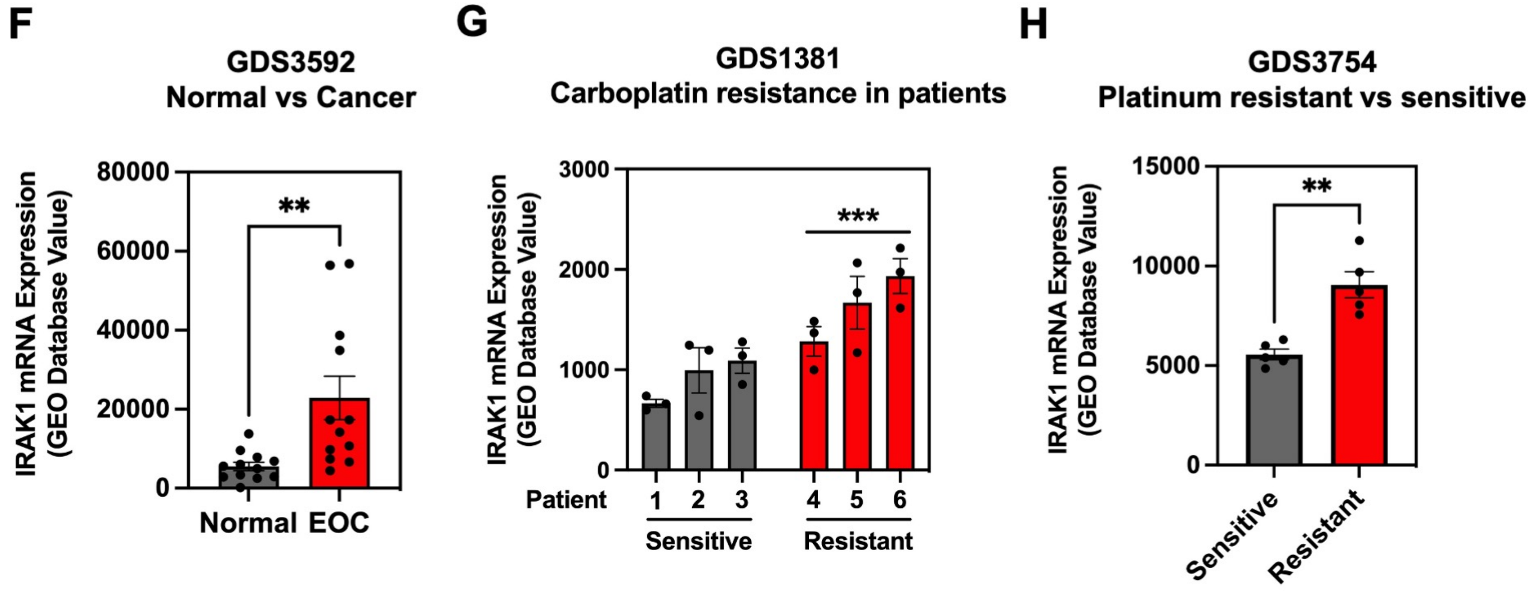

# Supplementary Figure 3

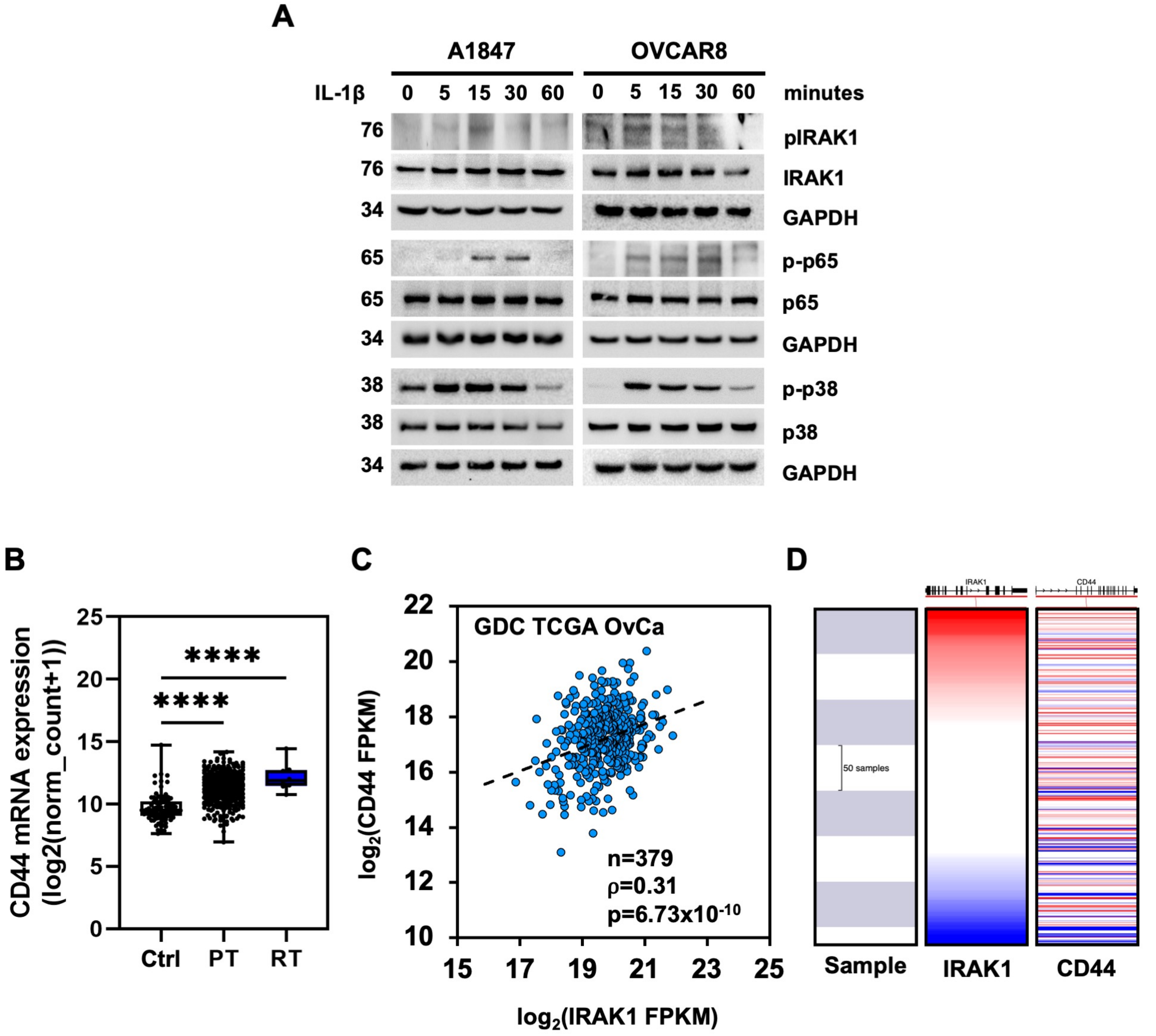

# Supplementary Figure 4

A

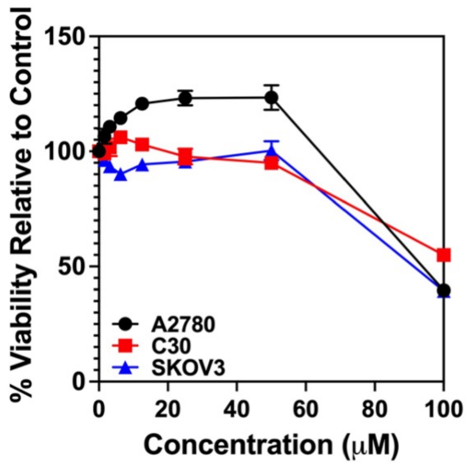


B

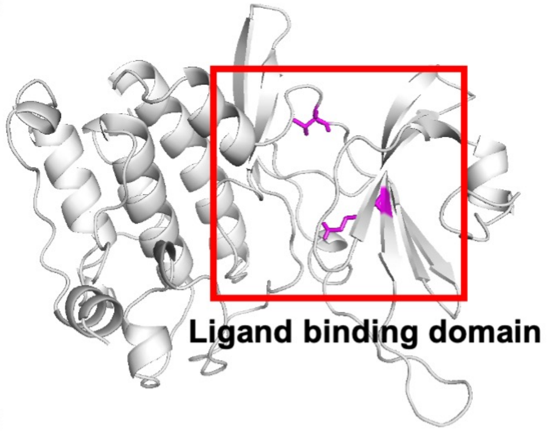


C

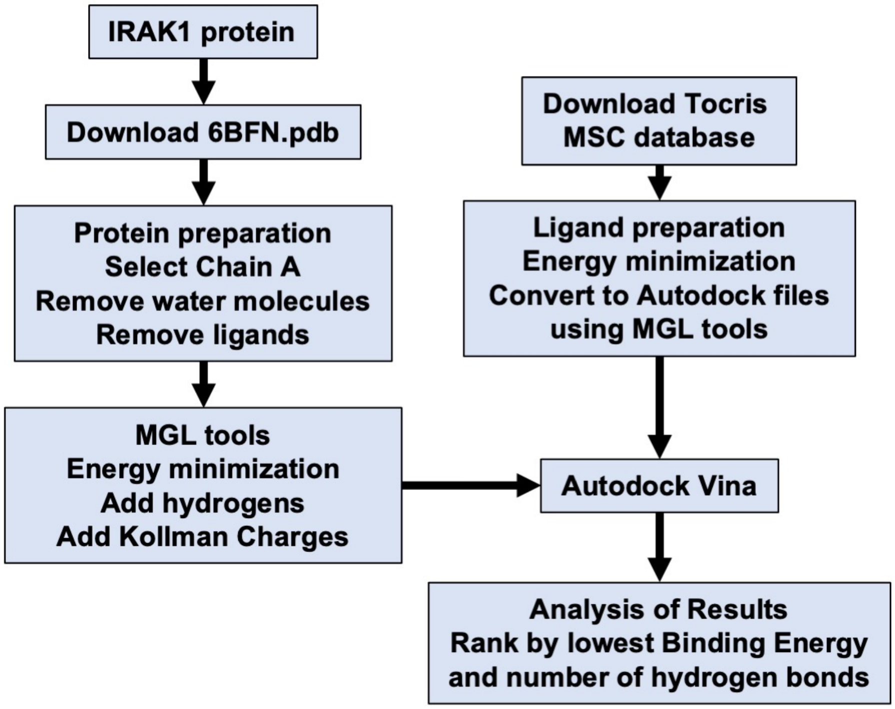


D

| Compound Name | Binding Energy (kcal/mol) |
|---|---|
| CW008 | -10.5 |
| TCS2210 | -10.4 |
| Troglitazone | -9.4 |
| H89 | -9.4 |
| Kartogenin | -9.2 |
| Indomethacin | -8.9 |
| Pioglitazone | -8.3 |
| Retinoic Acid | -8.2 |
| Zebularine | -7.2 |
| Dexamethasone | -7.0 |
| SB216763 | -6.9 |
| 5-azacytidine | -6.6 |
| Forskolin | -6.5 |
| Phorbol 12-myristate 13-acetate | -4.9 |
| Butyric Acid | -4.8 |

E

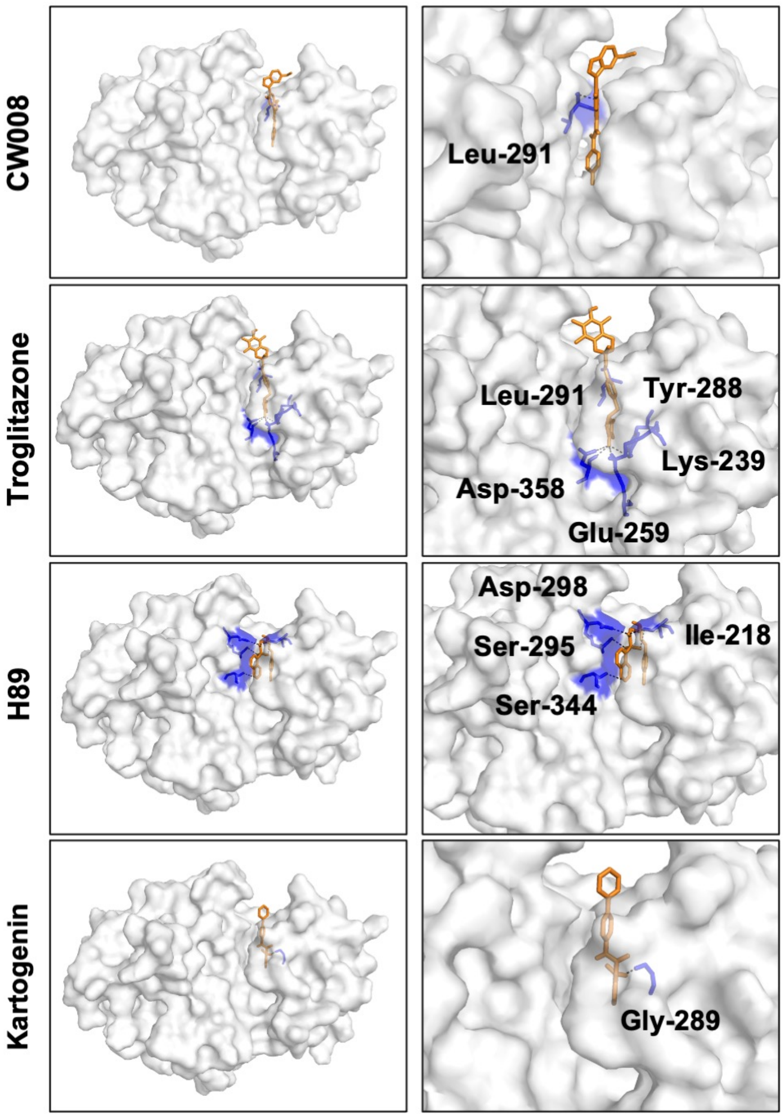

## Supplementary Figure 5

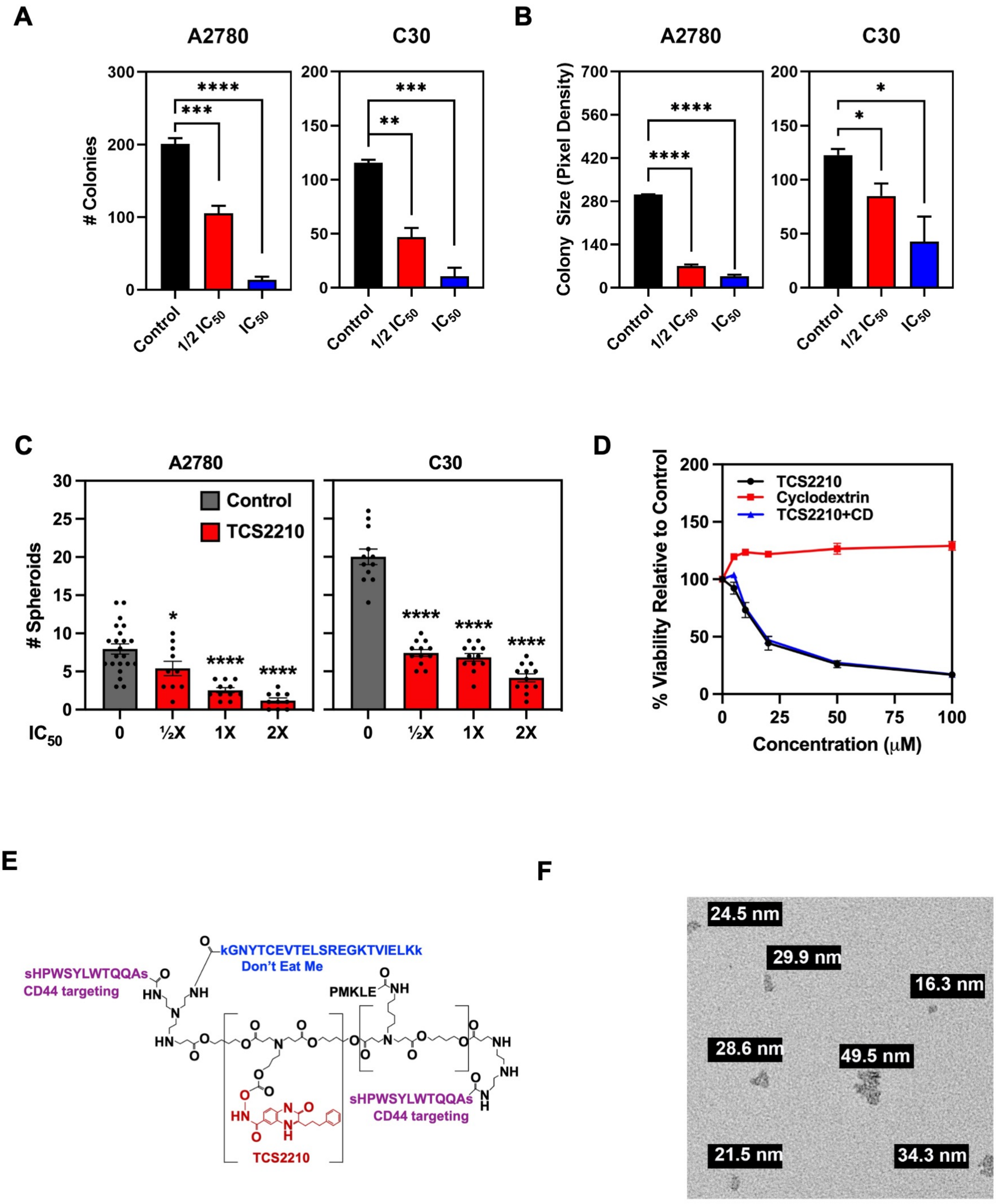

# Supplementary Figure 5

G

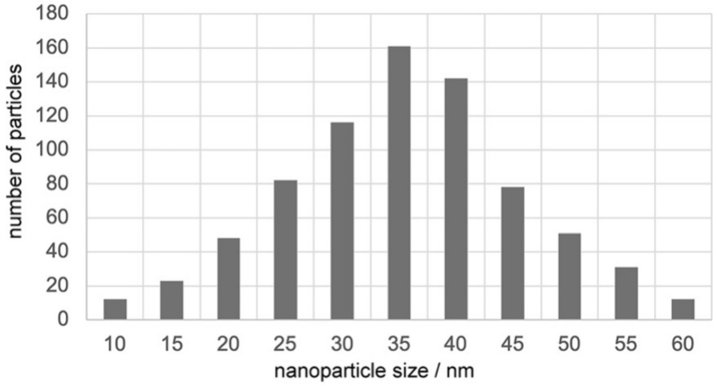


H

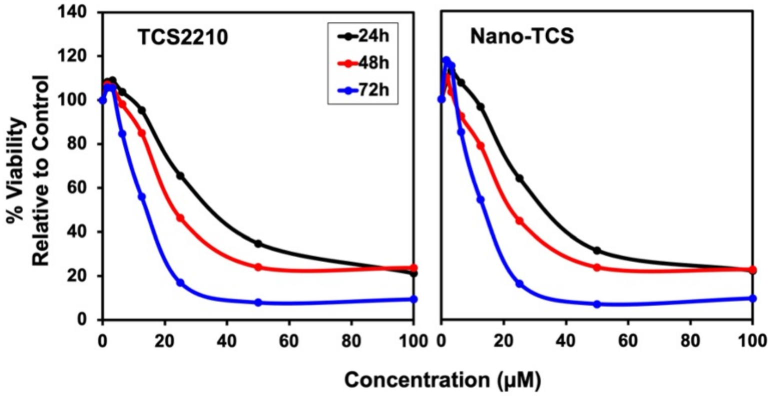

Supplement: Supplementary file 2 — Supplemental Figures [file 41419_2024_6717_MOESM2_ESM.pdf]
